# Withdrawal of antihypertensive medication: a systematic review

**DOI:** 10.1097/HJH.0000000000001405

**Published:** 2017-05-09

**Authors:** Veronika van der Wardt, Jennifer K. Harrison, Tomas Welsh, Simon Conroy, John Gladman

**Affiliations:** a Division of Rehabilitation and Ageing, School of Medicine, University of Nottingham, Nottingham; b Centre for Cognitive Ageing and Cognitive Epidemiology & The Alzheimer Scotland Dementia Research Centre, Royal Infirmary of Edinburgh, Edinburgh; c RICE (Research Institute for the Care of Older People), Royal United Hospital, Bath; d Department of Health Sciences, University of Leicester, Leicester, United Kingdom

**Keywords:** antihypertensive agents, antihypertensive medication, cessation, hypertension, withdrawal

## Abstract

Although antihypertensive medication is usually continued indefinitely, observations during wash-out phases in hypertension trials have shown that withdrawal of antihypertensive medication might be well tolerated to do in a considerable proportion of people. A systematic review was completed to determine the proportion of people remaining normotensive for 6 months or longer after cessation of antihypertensive therapy and to investigate the safety of withdrawal. The mean proportion adjusted for sample size of people remaining below each study's threshold for hypertension treatment was 0.38 at 6 months [95% confidence interval (CI) 0.37–0.49; 912 participants], 0.40 at 1 year (95% CI 0.40–0.40; 2640 participants) and 0.26 at 2 years or longer (95% CI 0.26–0.27; 1262 participants). Monotherapy, lower blood pressure before withdrawal and body weight were reported as predictors for successful withdrawal. Adverse events were more common in those who withdrew but were minor and included headache, joint pain, palpitations, oedema and a general feeling of being unwell. Prescribers should consider offering patients with well controlled hypertension a trial of withdrawal of antihypertensive treatment with subsequent regular blood pressure monitoring.

## INTRODUCTION

Antihypertensive medication is usually continued indefinitely, unless there are adverse effects that alter the original judgement that the potential benefits outweigh the potential harms. However, antihypertensive medication is withdrawn for other reasons, such as in preparation for trials of novel antihypertensive drugs. When this is done, it has been observed that some patients remain below the blood pressure (BP) threshold for restarting antihypertensive treatment (AHT). If this were to be sustained and well tolerated, attempted withdrawal of antihypertensive medication could be part of the routine management of hypertension – it would reduce the costs and risks of the use of antihypertensive drugs – presumably without losing the benefits in those who truly need them. Such attempted withdrawal might be particularly welcome for patients wishing to reduce their medication load, those deemed to be at high risk of problems from polypharmacy or patient groups in which there is uncertainty about the value of antihypertensive therapy – such as people with dementia.

For the planned withdrawal of antihypertensive therapy to be a worthwhile part of management, it would be necessary not only for a reasonable proportion of patients to be able to do so without needing to restart them, but they would need to remain below the threshold for restarting them for a considerable amount of time, and the process of the planned withdrawal of antihypertensive therapy would need to be safe. Planned withdrawal of antihypertensive therapy could be more efficiently targeted if there were reliable predictors of those who could and could not withdraw successfully and sustainably.

We undertook a systematic review of the literature describing studies in which antihypertensive therapy had been withdrawn to determine the proportion of people remaining within the threshold for restarting treatment for at least 6 months, predictors for successful and sustained withdrawal and the safety of doing so.

## METHOD

The systematic review was based on a predefined protocol (Prospero number CRD42016037130).

### Eligibility criteria

*Inclusion criteria:* original research articles and abstracts investigating withdrawal of antihypertensive medication in human adults; studies including people diagnosed with essential hypertension.

*Exclusion criteria:* animal studies, glaucoma and ocular hypertension studies, studies limited to hypertension in pregnancy, pulmonary hypertension studies, acute intercurrent illness, guanfacine studies (unless they include other antihypertensive medications analysed separately), systematic reviews, editorials, comments (unless on articles included in the review).

### Information sources

A meta-search was completed in Ovid using EMBASE (1974–30 April 2015), Medline (1946–4th week April 2015), Medline in Process and other nonindexed citations (30 April 2015), International Pharmaceutical Abstracts (1970–April 2015) and PsychINFO (1806–4th week April 2015).

### Search terms

Antihypertensive (exploded) AND Withdrawal OR cessation OR discontinuation OR stop^∗^

Filters were used where available to limit the search to studies of humans aged at least 19 years written in English.

#### Data selection

After deduplication, V.v.d.W. screened titles and abstracts based to identify eligible articles. Full texts for all eligible articles were obtained and assessed by two independent reviewers (V.v.d.W./T.W. or V.v.d.W./J.K.H.). When there was no consensus between reviewers, disagreement was resolved by discussion within the team.

#### Data extraction

The following data were extracted: author; year of publication; country; study design, sample characteristics, eligibility criteria, primary outcomes, antihypertensive medication before withdrawal, proportions remaining below the authors’ thresholds for recommencement of antihypertensive therapy at the time points reported in each study (i.e. successful withdrawal), factors predicting successful AHT withdrawal, adverse events or changes potentially leading to adverse events.

#### Risk of bias assessment

Depending on study design, the appropriate Critical Appraisal Skills Programme tool [1] was used to assess study quality. Quality assessment findings are summarized in the results section and reported for the individual studies in the tables in Supplementary content 1 and 2.

### Analyses

Our outcomes of interest were as follows:

1. The proportion of people remaining below the study's threshold for hypertension treatment (i.e. successful withdrawal).
2. The effects associated with withdrawal.

The proportion [and confidence intervals (CIs)] of people successfully withdrawn from AHT was identified or calculated on the basis of percentages for each study and summarized using means adjusted for sample size for 6 months (24–26 weeks), 1 year (52–60 weeks) and any longer follow-up periods (between 72 weeks and 6 years). If a study reported more than one result using different analyses, the lower proportion was used. If a study reported more than one result within a given analysis interval (e.g. for 2 and 3 years), the result for the longer follow-up period was used. Pearson's correlation coefficient between the year of publication and the proportion of people successfully withdrawn was calculated to investigate if there was a relationship between the historical context of the study (i.e. the hypertension guidelines at the time of the study) and the success rate of withdrawal.

Factors predicting successful withdrawal and adverse events were summarized in a narrative analysis; a quantitative meta-analysis was not possible due to the heterogeneity of parameters reported. Studies investigating adverse events or changes potentially leading to adverse events are tabulated in Supplementary digital content 1. A detailed analysis of changes potentially leading to adverse events is shown in Supplementary digital content 2. Risk and changes potentially leading to adverse events were structured by type of antihypertensive medication (if applicable).

## RESULTS

**FIGURE 1:**
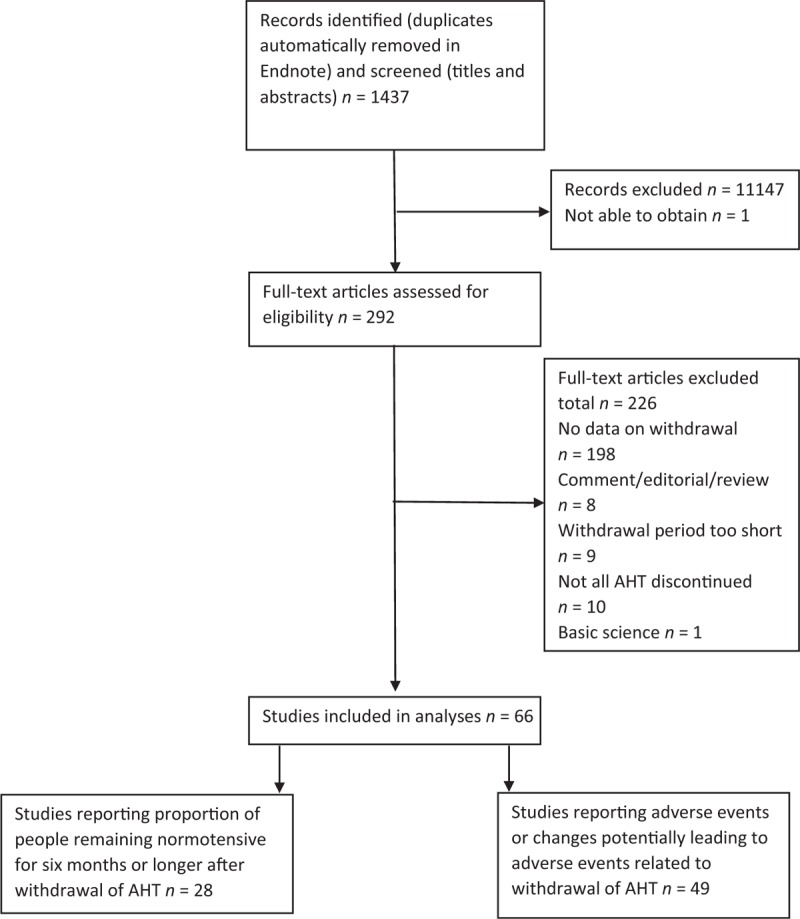
PRISMA flow diagram.

In total, 66 articles were included in the review (Fig. 1) with 28 studies reporting proportions of people within the threshold for treatment specified in the study for 6 months or longer after withdrawal of antihypertensive therapy (a description of the individual studies can be found in Supplementary digital content 1) and 49 studies reporting adverse events or changes potentially leading to adverse events related to withdrawal of antihypertensive medication (Supplementary digital content 2). Three studies [2–4] were not included in the analysis of the proportion of people within the threshold for treatment specified in the study. One reported a different analysis of an already included study [5], which resulted in a higher proportion of people within the threshold for treatment specified in the study [2], and two did not provide proportions but periods until hypertension returned [3,4]. For completeness of reporting, these studies were included in Supplementary digital content 2.

There was a significant negative relationship between the year of publication and the rate of successful withdrawal at 1 year (*r* = −0.52; *P* < 0.01; *n* = 26) with more recent studies showing lower proportions of successful withdrawal. There were no significant correlations between year of publication and rate of successful withdrawal at 6 months or 2 years (for 6 months: *r* = −0.44, *P* = 0.20, *n* = 10; for 2 years: *r* = −0.05, *P* = 0.86, *n* = 17).

### Quality assessment

Most studies were relatively small, with sample sizes of *n* less than 80. In the studies reporting the proportion of people within the threshold for treatment specified in the study, 15 out of 29 studies included sample sizes lower than *n* = 80. In the studies reporting adverse events or changes potentially leading to adverse events due to withdrawal of antihypertensive medication, 37 out of 49 studies included sample sizes lower than *n* = 80. Follow-up periods after withdrawal to assess adverse events were largely short term (29/49 studies reported adverse events for a follow-up of 1 month or shorter). The majority of studies were cohort studies, only a few were designed as randomized controlled trials (RCTs; 6/29 studies reporting the proportion of people within the threshold for treatment specified in the study were RCTs and 6/49 studies reporting adverse events or changes potentially leading to adverse events were RCTs).

#### Proportion remaining normotensive after AHT withdrawal

The analysis of studies reporting proportions of people remaining normotensive for 6 months or longer included 21 cohort studies and seven RCTs (Supplementary digital content 1). Thresholds for treatment differed across studies with thresholds for SBPs ranging from 135 to 200 mmHg and threshold for DBPs ranging from 85 to 129 mmHg. Three studies did not predefine a threshold but used a threshold depending on physician advice or patient decision [6–8]. The mean age reported in the studies ranged from 41 to 76 years. Out of the 28 studies, 17 did not specify the type of AHT withdrawn [3,6–22]. The remaining studies withdrew angiotensin-converting enzyme inhibitors [23], angiotensin II receptor antagonists [24], clonidine [23], beta blockers [4,23,25–27], calcium channel blockers [23,24,28], diuretics [2,4,5,21,25–27,29], reserpine [21] or vasodilators [4,21]. The follow-up interval ranged from 10 weeks to 6 years. Only three studies provided details regarding the withdrawal procedure [3,6,10] when multiple AHTs were withdrawn.

**FIGURE 2:**
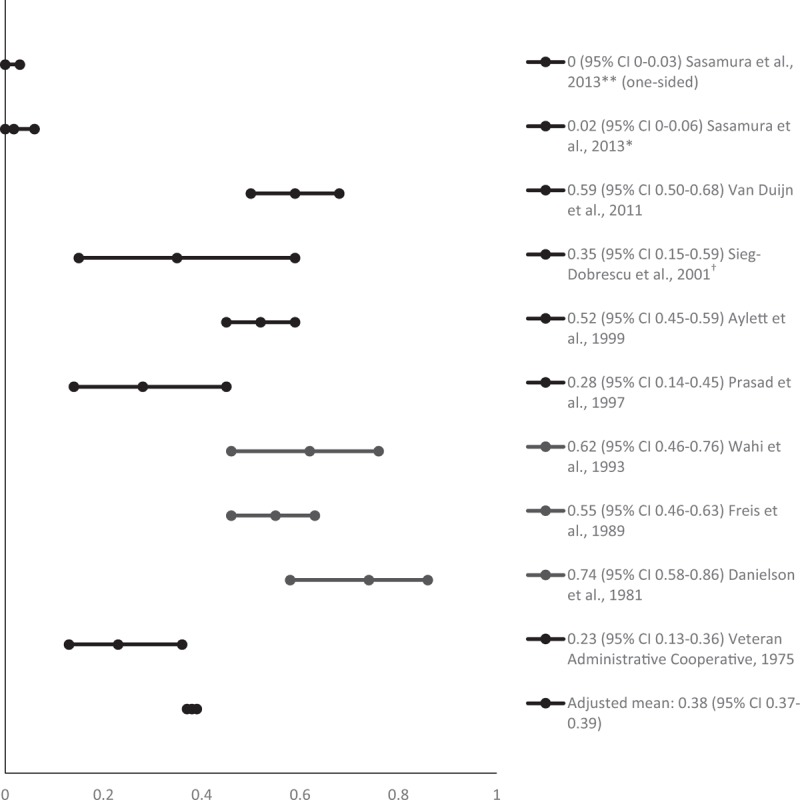
Proportion of people remaining normotensive at 6 months after antihypertensive treatment withdrawal. ^∗^For Candesartan group; ^∗∗^for Nifedipine group; ^†^at 24 weeks.

**FIGURE 3:**
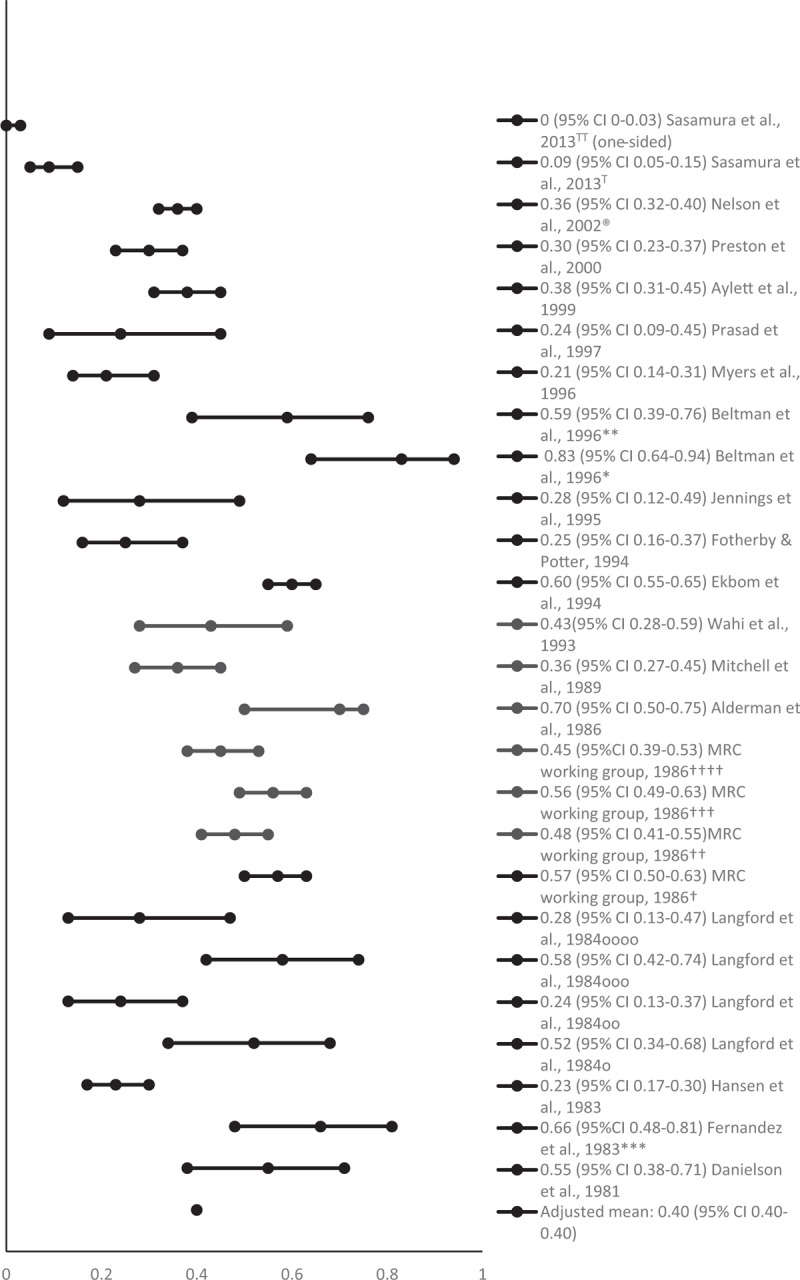
Proportion of people remaining normotensive at 1 year after antihypertensive treatment withdrawal. ^∗^Using office blood pressure; ^∗∗^using ambulatory blood pressure monitoring; ^∗∗∗^the study is reported in two articles with differing analyses: only results with lower percentage reported used (at 60 weeks); ^†^men, bendrofluazide group; ^††^men propranolol group; ^†††^women bendrofluazide group; ^††††^women propranolol group; the Nelson *et al.*, [3] study was not included as this reported a different analysis of the same study; ^T^For Candesartan group; ^TT^for Nifedipine group; ^o^overweight and mild HT; ^oo^overweight and severe HT; ^ooo^not overweight and mild HT; ^oooo^not overweight and severe HT.

**FIGURE 4:**
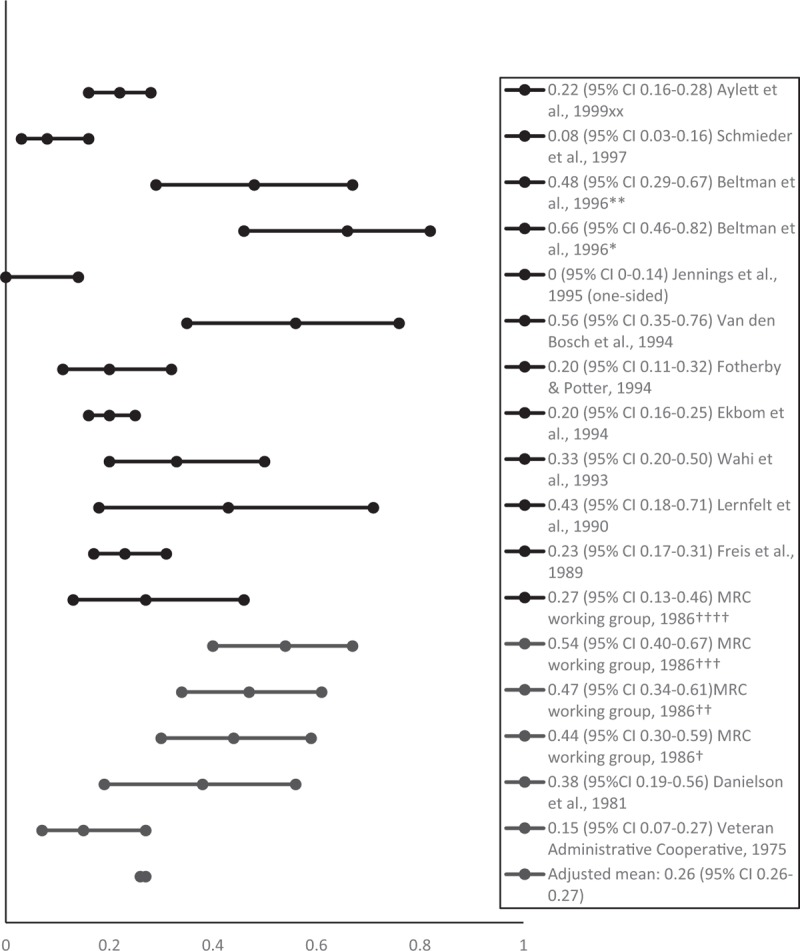
Proportion of people remaining normotensive at 2 years or longer after antihypertensive treatment withdrawal. ^xx^Based on the results for 3-year follow-up measurements; ^∗^using office blood pressure based on diastolic BP only; ^∗∗^using ambulatory blood pressure monitoring based on diastolic BP only; ^†^men, bendrofluazide group; ^††^men propranolol group; ^†††^women bendrofluazide group; ^††††^women propranolol group.

The mean proportion of people successfully withdrawn from AHT (adjusted for sample size) was 0.38 at 6 months (95% CI 0.37–0.49; 912 participants) [6,8,17,19,21,22,24,25,29], 0.40 at 1 year (95% CI 0.40–0.40; 2640 participants) [5–7,9–12,14–18,22,24–28] and 0.26 at 2 years or longer (95% CI 0.26–0.27; 1262 participants) [6,7,10,13,20–23,25–29]. Figures 2–4 show the proportions and 95% CIs for each included study and the mean proportions (adjusted for sample size) and 95% CIs for 6 months, 1 and 2 years or longer after AHT withdrawal. The majority of studies included middle-aged and older people. Due to the variety of measures of central tendency, a mean age could not be calculated.

### Factors associated with successful antihypertensive treatment withdrawal

Factors associated with successful AHT withdrawal were reported in 14 studies. The studies investigated a total of 18 parameters including, sex, age, ethnicity, height, body weight, low-dose AHT or monotherapy, severity HT at 5–6 years previous to study, BP levels before withdrawal, duration AHT, lower sodium levels, lower corpuscular volume, higher serum albumin, baseline alcohol or cigarette use, history of vascular disease, baseline of left ventricular mass index, baseline ECG voltage and cardiovascular risk factors. Only five studies reported all variables included in the statistical analysis, significant predictors and appropriate statistics [8,12,16,23,29]. Based on the available evidence, a meta-analysis was not possible. The studies suggest that monotherapy of AHT and lower BP before withdrawal predict successful withdrawal [7,8,10,15,16,23]. Although studies have also reported that body weight might predict successful withdrawal, the evidence is conflicted; although Fernandez *et al.* [5] and Nelson *et al.* [16] found that higher body weight and greater waist–hip ratio predicted remaining normotensive after withdrawal, the results of another cohort study suggested that lower BMI at baseline [10] was a significant predictor of normotension after AHT withdrawal.

#### What are the effects associated with withdrawal?

Studies investigating risks, that is adverse events and changes that could lead to adverse events are tabulated in Supplementary digital content 1. The risks included general adverse events as follows: changes in biochemistry, heart rate, pulse rate, haemodynamics, kidney function, left ventricular parameters, orthostatic hypotension and rebound hypertension (results reported in Supplementary digital content 2).

The review identified one single-drug RCT, which showed that there was no difference in adverse events at 1 month after withdrawal between those who stopped and those who continued renin inhibitor or angiotensin converting enzyme inhibitor treatment [30], although there were significantly more adverse events reported in the angiotensin-converting enzyme inhibitor groups compared with the renin inhibitor group.

Some studies withdrawing centrally acting AHT or a mixture of different hypertension treatments have shown some minor side effects such as general malaise, palpitations, joint pain, ankle oedema and headaches [22,27,30,31].

A large cohort study with a long follow-up period (5 years) demonstrated that those who had stopped antihypertensive medication and were not advised by their general practitioner to restart, had a lower risk of cardiovascular events and an equal mortality risk compared with those remaining hypertensive. However, it is important to note that patients who remained hypertensive included people being treated for hypertension as well as those in the placebo group of the STOP-Hypertension trial, which might have led to a higher risk of cardiovascular events [7].

## DISCUSSION

The adjusted mean for the proportions of people remaining normotensive after withdrawal of antihypertensive medication was 0.37 at 6 months and 0.26 at 2 years or longer. This suggests that one in four people could be successfully withdrawn from AHT for 2 years or longer. Monotherapy, lower BP before withdrawal and body weight might predict successful withdrawal. Adverse events were slightly more common in those who withdrew than in those who continued and but were minor and comprised headache, joint pain, palpitations, oedema and a general feeling of being unwell.

### Strength and limitations

Many of the studies identified were small, had a short follow-up period, used different thresholds for hypertension and did not identify potential long-term risks such as cardiovascular or cerebrovascular events. Nevertheless, we identified 17 largely consistent studies including over 1000 patients in total studied for 2 years or more to show that long-term withdrawal of antihypertensive therapy without the return of hypertension was possible in 26%. Although there will be concerns about the cessation of antihypertensive therapy, the study with one of the longest follow-up periods (5 years) indicated that those who had stopped AHT and were not advised by their general practitioner to restart AHT (withdrawal group), had a lower risk of cardiovascular events and an equal mortality risk compared with those either remaining on treatment or included in a placebo group [7]. We note that there was no consistent correlation between the year of publication and the chance of successful withdrawal of AHTs. An association was seen for studies reporting at 1 year after AHT withdrawal, but not for those reporting at 6 months or 2 years. If there is an association, a possible explanation for it could be that more modern studies used a combination of office measurements, ambulatory BP monitoring and home BP measurements [32] thereby reducing the proportion of patients who were over-diagnosed at outset. It is therefore possible that lower rates of successful withdrawal of AHTs will be seen in current practice than reported here. Our study found a limited amount of evidence about the effects of withdrawing some types of AHT such as renin inhibitors, calcium antagonists, vasodilators or hydrochloride, and so care must be taken in assuming that our findings apply to all antihypertensive agents. Most studies in this review included middle-aged and early older people and excluded those who had cerebrovascular or cardiovascular events, which means that our findings may not generalize to patients at high vascular risk.

Our findings here are consistent with our earlier preparatory review (a review of reviews), which indicated that antihypertensive medication can be withdrawn in 22–50% of people for 1 year or longer without hypertension returning [31].

Just as the current review did not identify serious risks to withdrawing AHT, similar results were found in the DANTE trial, which included people who were 75 years or older with mild cognitive deficits investigating the effects of AHT withdrawal [33]. The results of this study showed that there was no significant difference in adverse events between withdrawal and control groups at 4 months after withdrawal. The DANTE trial also confirmed the risk reduction of orthostatic hypotension due to stopping AHT [32], which was shown in one study included in this review.

## CONCLUSION

The current review of the available evidence shows that around one-quarter of mainly older patients on antihypertensive therapy can withdraw such medication without return of hypertension for 2 or more years. Doing so has a small risk of minor side effects and if successful may be associated with a lower cardiovascular risk in those with lower BPs at the point of withdrawal. On this basis, it seems reasonable for prescribers to consider using this evidence to offer patients with well controlled hypertension a trial of withdrawal of their antihypertensive medication. From a practical perspective, the reporting of procedures regarding the withdrawal of AHT was limited in the included articles; however, details for planned AHT withdrawal have been published elsewhere [34]. Because of the limitations of the evidence, it should be noted that the safety of this approach is not known for people at high cardiovascular risk. Our findings indicate that attempting to withdraw AHT will be more successful in those with lower BPs and on single agents. However, given that three quarters of patients attempting withdrawal will return to BPs that would indicate the need to offer restarting antihypertensive medication, stopping medication does not mean an end to monitoring. Nevertheless, some patients may wish to attempt, a trial of withdrawal – particularly those for whom there is already doubt about the benefits of antihypertensive therapy, such as in people with dementia [35,36].

Further research would be wise to determine what proportion of people agree to trials of withdrawal of their antihypertensive therapy and the reasons for their choices. Studies should examine the short and long-term benefits and harms observed when doing so, particularly in groups of patients in which deprescribing might be considered. Research into programmes of deprescribing not focussed solely on AHTs, for example in polypharmacy, should take these findings into account when designing their interventions.

## ACKNOWLEDGEMENTS

The current article presents independent research funded by the National Institute for Health Research (NIHR) under its Research for Patient Benefit (RfPB) Programme (grant reference number PB-PG-1112-29070). The views expressed are those of the authors and not necessarily those of the NHS, the NIHR or the Department of Health.

J.G. was also funded by NIHR CLAHRC East Midlands. V.v.d.W., J.G., S.C. and J.K.H. report receiving a grant for this work from the National Institute of Health Research/United Kingdom.

### Conflicts of interest

There are no conflicts of interest.

## Supplementary Material

Supplemental Digital Content

## Reviewers’ Summary Evaluations

### Reviewer 2

Withdrawal of antihypertensive therapy is a contentious issue. This systematic review provides a good summary of the evidence to date. Although the studies that have been analysed varied in size and protocol design, a consistent finding is that a significant proportion of people who were well controlled on antihypertensive therapy did not revert to hypertensive BP levels after their drugs were stopped. A well conducted systematic review such as this can only be hypothesis-generating and what would be needed is a large trial with contemporary methods of blood pressure measurement and more precise phenotyping of patients.

### Reviewer 3

Studies included in this systematic review on the effects of withdrawal of antihypertensive therapy in hypertensive patients were published between 1975 and 2013. During this time, research criteria became more stringent, greater emphasis was placed on randomized controlled trials, and thresholds and targets of antihypertensive therapy have become more evidence-based. Furthermore, ambulatory and home BP became important in the management of hypertension, so that it is highly likely that, without out-of-office BP, the data analyzed in this study were influenced by white-coat hypertension. Therefore, the review is mainly of historic interest and new studies are warranted, taking into account contemporary guidelines for the management of hypertension.
