# Supplementary material for: Withdrawal of antihypertensive medication: a systematic review
Source: J Hypertens. 2017 May 9;35(9):1742–9. doi: 10.1097/HJH.0000000000001405 (PMC5548513; doi:10.1097/HJH.0000000000001405)
Supplement: Supplemental Digital Content [file jhype-35-1742-s001.docx]

Supplementary digital content 1: Detailed overview of studies reporting proportions of people remaining normotensive for six months or longer

| **Author Date Country** | **Study design** | **Threshold for hypertension in mmHg** | **Sample size and age** | **Eligibility criteria** | **Primary outcomes of study** | **AHT used before withdrawal** | **% people normoten-sive after 6 months** | **% people normotensive after >6 months** | **Factors predictive of successful for 6 months or longer withdrawal** | **CASP appraisal - limitations of studies** |
| --- | --- | --- | --- | --- | --- | --- | --- | --- | --- | --- |
| Alderman et al., 1986; USA [1] | Prospective cohort study with a 1 year follow up | Under 65 years old: BP ≥160/95 mmHg; over 65 years old: BP≥165/95 mmHg | n=66; mean age = 55.7 (SD 8.3) years; 27% men; | Inclusion: taking AHT at time of screening; mean of 2 or more readings pre-treatment ≥ 160/95 mmHg; use AHT for another 6 months following screening; no contra-indication to AHT withdrawal; possibility to participate in 2 year follow -up; for people under 65 years: 2 BP readings within 6 months <140/85 mmHg, for over 65 years old: two BP readings in 6 months <150/90 mmHg | Number of participants remaining below threshold BP | Any | - | At 1 year: 70% (44 out of 63); of those remaining normotensive at year 1, 35 were followed up after 2 years: 51%(18 out of 35); those remaining normotensive at year 2, 35 were followed up after 3 years: 88% (15 out of 17) | Although there was no difference in BP between those successfully and unsuccessfully withdrawn at the point of withdrawal, all subsequent mean BP readings of patients remaining normotensive were sig. lower than the mean BP readings of those whose AHT needed to be reinstated; | Small sample size; |
| Aylett et al., 1999; UK [2] | Longitudinal observational study with a 3 year follow-up | Determined by the patient's practitioner on the basis of individual risk profiles | n=196; age range: 40-69 years; sex not reported; | Inclusion: people with HT taking AHT; exclusion: poorly controlled (BP>160/90 mmHg); on AHT for less than 2 years; had major cardio- vascular event within last year; indication for AHT in addition to HT; had a previous relapse of HT after stopping AHT | Proportion of patients remaining below threshold BP | Any | 52% | At 12 months: 38%; at 24 months: 23%; at 36 months: 22% | Male sex | Very large sample size; sample; demographic characteristics not well reported |
| Beltman et al., 1996; Netherlands [3] | Observational study following an AHT RCT with 1 year follow up | Ambulatory BP of 135/85 | n=29; mean age= 51 (SE 2); men n=18; | Inclusion: completion of RCT (one year of treatment with amlodipine or lisinopril); dias BP<90 mmHg during RCT | Proportion of patients remaining below threshold BP | Amlodipine or lisinopril | - | Using ambulatory BP with a dias cut-off only: at week 60: 59% (17 out of 29); at week 104: 48% (14 out of 29); using seated office BP with a dias cut-off only: at week 60: 83% (24 out of 29); at week 104: 66% (19 out of 29); | - | Small sample size; |
| Danielson et al., 1981; Sweden [4] | Prospective cohort withdrawal study | Between 155/95 and 180/110 depending on age and risk factors | n=47; mean age = 42 years (SD 11); men n = 21; | Inclusion: essential hypertension state 1 for not less than 1 year; well controlled HT for > 6 months | Number of patients remaining normo-tensive without returning to AHT | Hydrochlorothiazide, beta-blockers or a combi-nation of both | 74% (31 out of 42) | At 1 year: 55% (22 out of 40); at 2 years: 36% (10 out of 28) | - | Population not well described; small sample size; |
| Ekbom et al., 1994; Sweden [5] | Observational study with a 5 year follow-up including participants of the wash-out phase of the STOP -Hypertension trial | No level set, restarting AHT depended on GP advice | n=333; mean age= 75.2 (SD 3.8; range 70-84); 32% men | Inclusion: hypertension; between 70 to 84 years old | Time until AHT was re-started | Any | - | At 1 year: 60%; at 5 years: 20%; | Monotherapy, low dose and low BP level before withdrawal were significant predictors for successful withdrawal; age, sex, duration of drug treatment before withdrawal and low BP levels before patient received treatment for the first time were not significant predictors | Very large sample size; sample; no pre-set BP threshold; |
| Fernandez et al., 1982; Canada [6] | Prospective cohort study: 60 week follow -up for those remaining normotensive after wash-out phase for a cross over study; | 140/95 | n=24; mean age = 43.5 (SD 11.6), men n = 17; | Inclusion: uncomplicated essential HT; HT diagnosis with 3 supine and 3 erect BP readings of dias BP > 95 mmHg and sys BP > 140 mmHg; exclusion: cardiovascular disease, grade III or IV retinopathy, liver or renal impairment, diabetes, hypokalaemia or any condition that did not justify inclusion | BP levels after withdrawal of AHT | Hydrochlorothiazide and loop diuretic (MK-196) | - | At 48 weeks: 100%; at 60 weeks: 75% (18 out of 24) | - | Small sample size; likely to be the same study as Fernandez 1983 though results are different; |
| Fernandez et al., 1983; Canada [7] | Prospective cohort study; AHT discontinued for cross-over study and those remaining normotensive followed up for 60 weeks; | 140/95 | n=35; age range : 21-65 years; | Inclusion: uncomplicated essential HT; HT diagnosis with 3 supine and 3 erect BP readings of dias BP > 95 mmHg and sys BP > 140 mmHg; exclusion: cardiovascular disease, grade III or IV retinopathy, liver or renal impairment, diabetes, hypokalaemia or any condition that did not justify inclusion | Number of patients remaining normotensive without AHT | Hydrochlorothiazide and loop diuretic (MK-196) | - | At 60 weeks: 66% (23 out of 35) | Comparison of a wide range of biomarkers that separated those who remained normotensive from those redeveloping hypertension. Those in the normotensive group had lower serum sodium levels, lower mean corpuscular volume, higher serum albumin and higher body weight | Small sample size, in particular for this type of analysis; likely to be the same study as Fernandez 1983 though results are slightly different; |
| Fotherby & Potter, 1994; UK [8] | Prospective cohort study with 1 year follow-up | 160/90 on 2 consecutive visits | n = 74; mean age 76 years (SD 5; range 65-84); 47 % men; | Inclusion: fit, hypertensive, on AHT for > 1 year, age ≥ 65; exclusion: symptoms of angina or congestive heart failure, history of myocardial infarction or stroke within preceding 6 months, renal impairment, on other medication sig affecting BP, other diseases that would sig affect survival | Proportion of people who remained normotensive at 12 months after withdrawal | Any | - | At 12 months: 25% n=20); of n=64 available at 2 years follow-up, 20% (n= 13) remained normotensive | No sig difference between those remaining normotensive at 1 year and those restarting AHT in age, sex number of AHT, alcohol or cigarette use, history of vascular disease, treatment duration, LV mass index, clinic or mean 24 h dias BP; less AHT, lower sys BP, ECG voltage and BMI at baseline were sig predictors of remaining normotensive | Small sample size; study included lifestyle advice (but no further intervention); |
| Freis et al, 1989; USA [9] | RCT comparing AHT reduction, AHT withdrawal and control group followed up for up to 30 months | Dias BP 85-90 depending on previously or newly diagnosed; no threshold for systolic BP | total n=606; AHT discontinued n = 137; mean age = 56.9 years; all men; | Inclusion: previously diagnosed with a dias BP was <115 mmHg and newly diagnosed patients with a dias BP between 90-95 mmHg; exclusion: malignant HT, haemorrhagic stroke, recent myocardial infarction, history of alcoholism or drug abuse, contraindication to study; | Number of participants who maintained control BP | Hydrochlorothiazide (HCTZ) | 55% (of those discontinueing low dose of HCTZ) | At 18 months: 35%; at 30 months: 23% | Patients maintaining BP control had lower BP at baseline | Very large sample size; only low dose HCTZ discontinued |
| Hansen et al., 1983; Denmark [10] | Prospective cohort withdrawal study with 1 year follow up | For inclusion: diastolic BP 105-110 (depending on age); restart of AHT decided by GP | n = 169; over 50 years old; sex not reported | Inclusion: history of hypertension; | Number of participants not back on AHT | Any | - | At 12 months: 25% (n = 43) remained below a dias BP level of 110 mmHg; 23% (n = 39) remained below as dias BP level of 100 mmHg | - | Large sample size; recruitment and demographics of participants not well reported; |
| Herlitz et al., 1988; Sweden [11] | Cohort withdrawal study | 170/105 | n=22; mean age = 56 years; all men | Inclusion: regular visits to a HT Unit for 7 years; mild to moderate HT with dias BP levels ≥ 100 mmHg at first 3 visits | Time after withdrawal until HT returned | Beta blockers, beta blockers and HCTZ, or beta blockers, HCTZ and hydralazine | - | 50% remained normotensive (slow return group) for a mean of 240 days (range 65-578 days) | There was no difference in pre-treatment BP between rapid and slow return to HT groups; there was a difference in number of AHT used with more AHT used in the rapid return group | Small sample size; only men included; |
| Jennings et al., 1995; Australia [12] | Prospective cohort study with 2 year follow-up | Dias BP: 95mmHg on two successive visits | n=25, mean age = 51.3 (SEM 2.3); male/female ratio not reported | Inclusion: AHT for > 1 year; | Percentage normotensive at 12 months | Beta blockers and thiazide diuretic | - | At 10 weeks: 72%; at 20 weeks: 60%; at 12 months: 28%; at 24 months: 0% | On AHT for > 1 year; left ventricular hypertrophy assessed as posterior wall thickness ; | No sys threshold value used; small sample size; demographics not well reported; |
| Langford et al., 1984; USA [13] | RCT; participants were stratified into an obese and not obese group and randomised into dietary intervention or no intervention and AHT withdrawal or no withdrawal; only AHT withdrawal groups without lifestyle intervention included in review analysis | Medication restarted if dias BP 95-99 mmHg on 3 occasions within 3 months, or if 2 diastolic BP readings were in the 100-104 mmHg rage in a one month period, or if at any time diastolic BP rose to 105 mmHg or higher | n=496; age range: 30-69 years; sex not reported; overweight control group n = 89; not overweight control group n= 70; | Inclusion: active controlled participants of the previous Stepped Care HT Detection and follow-up programme; had no sys BP> 180 mmHg; average dias BP < 95 mmHg during past year; average of last 2 dias BP readings was < 95 and neither was > 95 mmHg | Number of people off AHT after 56 weeks | Any | - | Only control groups are reported here as the other groups used lifestyle interventions; follow up at 56 weeks: overweight: for mild HT 52% (19 out of 37); for severe HT (diastolic BP> 105 mmHg) 24% (12 out of 52); not overweight: for mild HT 58% (24 out of 41); for severe HT 28% (8 out of 29) | If participants had been diagnosed with severe HT 5-6 years previously, their relative odds of success to withdraw medication were 0.38 compared to those who had not have severe HT. If they were taking 2 drugs at start of study, odds for successful withdrawal were 0.47 compared to those taking one drug; they were taking 3 or more drugs at start of study, odds for successful withdrawal were 0.23 compared to those taking one drug; there was not sig. effect of age, race, sex, smoking status, overweight or study centre on relapse rate | Very large sample size; |
| Lernfelt et al; 1990; Sweden [14] | Prospective cohort study with 2 year follow-up | 200/105 | n=25; age = 70; 10 men (40%) | Inclusion: part of the '70 year old people in Gothenburg, Sweden' study; using AHT; exclusion: BP> 175/95 mmHg; cardiovascular disease; EEG abnormalities; increased heart volume; other serious diseases | Percentage of people who completed 2 years follow-up without treatment reinstated | Any | 85% (17 out of 20); | At 2 years: 43% (six out of 14) | - | Small sample size; |
| Medical Research Council Working Party on mild HT, 1986; UK [15] | Withdrawal RCT following RCT assessing treatment with propranolol or bendrofluazide | Dias BP > 90 mmHg | Men bendrofluazide (BF) group: 12 months: n=205; 24 months: n=50; propranolol (PP) group: at 12 months n=201; at 24 months: n=55; placebo: at 12 months: n=292; at 24 months: n=59; Women: BF group: 12 months: n=212; 24 months: n=54; PP: at 12 months n=165; at 24 months: n=33; placebo: at 12 months: n=290; at 24 months: n=66; | Inclusion: age 35-64 years; diastolic BP 90-109 mmHg; completion of medication trial | Rates of successful withdrawal of AHT at 3, 12 and 24 months | Bendrofluazide or propranolol | - | Men: BF group: 12 months: 56.5%; 24 months: 44%; PP group: at 12 months 48.2%; at 24 months: 47.3%; placebo: at 12 months: 51.8%; at 24 months: 42.4%; Women: BF group: 12 months: 56.3%; 24 months: 53.7%; PP group: at 12 months 45.2%; at 24 months: 27.3%; placebo: at 12 months: 52.7%; at 24 months: 40% | Following propranolol treatment: in older people BP levels tended to increase less quickly, this effect was not observed following bendrofluazide treatment or in those in the placebo group; after stopping bendrofluazide, sys BP levels rose more quickly in men (but not women) with increased entry levels; this effect was not seen when stopping propranolol; | Very large sample size; no sys BP threshold values |
| Mitchell et al., 1989; Canada [16] | Prospective cohort study with 1 year follow-up | Dias BP of second and third reading < 120; if average between 105-119, new visit one week later: if BP> 105 mmHg participant withdrawn from study | n=107; mean age= 51 years; 78% men | Inclusion: 30-70 years old; on AHT of 2 or less units with 1 unit being equivalent to 50 mg of hydrochlorothiazide; under good BP control (dias BP<105 during last 4 visits); no evidence of target organ disease; no contraindication to nadolol; no history of a psychotic disorder, diabetes, asthma or chronic obstructive lung disease | Number of participants remaining below threshold BP | Any | - | At 12 months: 36% (38 out of 107) | Sex was not predictive of successful withdrawal; lower standing dias.BP at study entry and total months of BP control was predictive of remaining normotensive; | No threshold values for sys BP; |
| Myers et al., 1996; Canada [17] | Cohort withdrawal study | Restart of AHT if mean of 2 ambulatory BP recordings exceeding 160/95; | n=98; mean age: 62 years (SD 2; range 21-80); men n = 36; | Inclusion: receiving AHT exclusion: target organ damage, secondary HT, cardiac arrhythmias, receiving other medication affecting their BP; if mean ambulatory sys BP ≥160 or dias BP ≥ 95 mmHg; if AHT had been changed in the last 3 months | Proportion of patients remaining off treatment at 1 year | Any | - | At 1 year: with a 160/95 cut-off 51% (50 out of 98); with a 150/90 cut-off 42% (41 out of 98); with a 140/90 cut-off 21% (21 out of 98) | Age, gender, intensity of AHT, and family physician BP readings were not related to successful withdrawal; diastolic BP taken by the research nurse and by ambulatory monitor at baseline was sig. higher in those who returned to drug therapy; | Well reported analysis with different cut-off values |
| Nelson et al., 2002; Australia [18] | Prospective cohort study with 12 months follow-up (extended run-in phase of AHT trial) | 160/90 | n=503; age range: 65-84 years; sex of total group not reported; | Inclusion: treatment with AHT, participant in the 2nd Australian national blood pressure study but BP remaining normotensive in run-in phase | Proportion of patients remaining below threshold BP | Any | - | At 12 months: 36% remained normotensive (181 out of 503) | Lower systolic BP, younger age, greater waist-hip ratio and use of a single AHT were sig. predictors for remaining normotensive; [not clear which variables were entered into analysis] | Very large sample size; |
| Nelson et al., 2003; Australia [19] | Wash-out cohort study preceding RCT | 160/90 | n = 6291; mean age = 71.9; 44.0% men; | Inclusion: between 65 and 84 years, receiving AHT; exclusion determined by GP: mostly those with pre-existing cardiovascular complications, difficulty to manage patients, those not willing to participate or those for who GP suggested to continue AHT | Patient characteristics of those remaining normotensive after AHT withdrawal | Any | - | n= 1228 remained normotensive (<160/90) for a median of 4 weeks (0-76 weeks, the longest period to return to hypertension was 108 weeks (1 patient) | Lower sys and dias BP levels before withdrawal, younger age, being on a single AHT; using the lower threshold of 140/90, the same predictors were identified with exception of younger age | Very large sample size; not clear which variables were entered into analysis of factors that predict remaining normotensive |
| Prasad et al, 1997; UK [20] | Prospective cohort study with 1 year follow-up | Daytime ABPM BP > 150/90 | n= 25; median age = 64 (range 51-82); 17 women, 19 men | Inclusion: on AHT monotherapy; exclusion: ischaemic heart disease, cerebrovascular disease, peripheral vascular disease, diabetes, renal impairment, secondary HT; | Proportion of people who remained normotensive at 52 weeks after withdrawal | Any | At 6 months: 28% (7 out of 25) | At 1 year: 24% (6 out of 25) | - | Small sample size; |
| Preston et al., 2000; USA [21] | RCT including active treatment and placebo groups with 1 year follow-up of the placebo group | Dias BP>95 | n = 1292; active treatment group: n = 1105; placebo group n = 187; no mean age reported; all men; | Inclusion: age > 21 years, reasonable expectation that dias BP would be between 95 and 105 mmHg with placebo; exclusion: secondary HT | Response to placebo at 1 year follow up and effect of race and age on response | Not reported | - | All placebo at 1 year: 30% (57 out of 187); | No difference between treatment and placebo groups regarding resting pulse rate, age or baseline laboratory values (haematocrit, serum electrolyte, cholesterol, triglyceride or 24 h urine sodium or potassium excretion levels) | Very large sample size; sample not well described; no sys BP threshold value set; |
| Sasamura et al., 2013; Japan [22] | RCT comparing withdrawal of 2 AHTs | If 1 of 4 criteria were reached: 1) office sys BP > 140 and/or dias BP >90 at 2 consecutive visits; 2) office sys BP>160 and/or dias BP > 100 at 1 visit; 3) mean weekly home sys BP > 140 and/or dias BP> 90 on 2 consecutive visits; 4) mean weekly home sys BP> 160 and/or dias BP> 100 at any 1 week | n=244; Candesartan group n = 124; mean age=51.1 (SD 6) years; men n = 75; nifedipine group n = 120; mean age= 51.3 (SD 5.7) years; men n = 79; | Inclusion: age 30-59 with stage 1 HT; family history of HT; no prior pharmacological treatment for > 3 months prior to study; exclusion: secondary HT, plans for pregnancy, diabetes, renal dysfunction, cardiovascular disease, severe liver dysfunction, malignancies or otherwise judged inappropriate | Survival until recurrence of HT | Candesartan or nifedipine | Candesartan group: 1.8%; nifedipine group: 0% | At 12 months: candesartan group: 0.9%; nifedipine group: 0% | - | Very large sample size; limited generalisability due to inclusion criterion 'family history of HT'; |
| Sieg-Dobrescu et al., 2001; Switzerland [23] | Withdrawal RCT with 24 week follow-up | 140/90 | Withdrawal group: mean n = 20; mean age = 48 years (SD 14.3); men n = 10; treatment group: n = 20; mean age = 47 years (SD 13.2); men n = 11 | Inclusion: receiving AHT for at least previous 6 months, BP < 140/90 mmHg; exclusion: requiring treatment for hypercholesterolemia, diabetes, angina pectoris or congestive heart failure, previous cardiovascular event | BP levels after withdrawal | Any | [at 24 weeks: 35% (7 out of 20)] | - | - | Very small sample size; |
| Schmieder et al., 1997; Germany [24] | Prospective cohort study with 6 months intensive treatment, followed by treatment under the supervision of GP; after 6 years patients were followed up | 160/95 and 140/90 | n= 88; at baseline mean age = 42.6 (SD 7.2) years | Civil servants; those included were male, not receiving AHT prior to the study and had no evidence of secondary HT; only those with BP levels > 160/95 mmHg were included | Percentage of those who have been withdrawn from AHT and predictors for recurrence of HT | Beta blockers, calcium entry blockers, angiotensin-converting enzyme inhibitors or clonidine | - | Follow up was after 6 years: with 160/95 mmHg cut-off: 17% (15 out of 88); with 140/90 mmHg cut-off: 8% (7 out of 88) | Lower screening dias BP; lower screening rest sys and dias BP; high cardiac output at rest, low total peripheral resistance at rest; cardiac output and increase in cardiac output during mental stress, increase in heartrate during exercise test were higher in patients who returned to HT; low echocardiographic left ventricular mass and short duration of HT were associated with normotension at follow-up; age, height, initial weight, weight after 6 years, coexistence of other cardiovascular risk factors did not predict successful withdrawal | Only men who had previously not received AHT were included |
| Van den Bosch et al., 1994; Netherlands [25] | Prospective cohort study with 5 year follow up | Dias BP: 100 | n = 25, mean age = 56.2 (range 39-81 years) | Inclusion: monotherapy, dias BP <85 mmHg on 3 or more readings, no other reason to continue AHT such as co-morbidities | Number of participants remaining below threshold BP | Any | - | At 5 years: 56% (14 out of 25) | No difference between groups regarding BP levels at diagnosis or start of withdrawal, or regarding history of HT (years since being diagnosed) | Small sample size; sample not well described; no sys BP threshold value set; |
| Van Duijn et al., 2011; Netherlands [26] | Prospective cohort study with 6 months follow up | Low cardiovascular risk and patient's decision to stop or re-start AHT | n= 135; age: 25-75 years; | Inclusion: no known target organ damage, receiving AHT and/or medication for hypercholesterolaemia; exclusion: cardiovascular disease, taking drugs for other indications than HT or hypercholeserolaemia; | Attendance for re-evaluation | Any | 59% (80 out of 135) | - | Those who used more than 2 oral drugs were more likely to restart medication, other characteristics (sex, age, smoking and family history of cardiovascular risk) were not predictive; | Reasons to re-start medication reported in combination with withdrawal of medication for hyperchole-sterolaemia; |
| Veterans Administration Cooperative Study Group on Antihypertensive Agents, 1975; USA [27] | RCT including continued treatment and withdrawal arm with 72 week follow up | Dias BP > 129 at one visit or dias BP > 114 at any 2 visits or dias BP > 104 at any 3 visits or dias BP > 94 at any 5 visits or any major cardiovascular complications | Withdrawal group: n = 60 ; mean age = 52.2 years; treated group: n=26; mean age 52.8 years; all men | Inclusion: participant of previous AHT trial; dias BP for 2 preceding visits ≤ 90 mmHg; no dias BP > 95 mmHg during any of the 3 preceding visits; average dias BP during preceding 12 months ≤95 mmHg | Number of participants remaining below threshold BP | Hydrochlorothiazide, reserpine and hydralazine | 23% (14 out of 60) | At 72 weeks: 15% (9 out of 60) | Lower BP levels prior to entrance into AHT trial (preceding withdrawal trial); no correlation with age; | Small sample size; no sys BP threshold value set; |
| Wahi et al., 1993; India [28] | Prospective cohort study with a 1 year follow up | 160/90 at 2 readings or 200/110 at one reading | n = 42; mean age = 40.7; (range 22 to 65); 30 men; | Inclusion: 15-65 years; no secondary HT; on AHT; had BP on 2 occasions before start of AHT; at least 6 months of AHT; no contraindication to AHT withdrawal; possibility to follow up for a minimum of 1 year; BP averaging less than 140/85mmHg in 6 months before withdrawal and 140/90 mmHg on start of withdrawal | Number of participants remaining below threshold BP | Any | 61.9% (26 out of 42) | at 12 months: 42.8% (18 out of 42); at 100 weeks 33% (14 out of 42) | Being younger, having milder HT, a shorter period of diagnosed HT and using monotherapy was related to remaining drug free for longer | Small sample size; sample not well described; |

AHT: antihypertensive treatment; dias: diastolic; HT: hypertension; sys: systolic; sig: significant;

References

1. Alderman, M.H., et al., *Antihypertensive drug therapy withdrawal in a general population.* Archives of Internal Medicine, 1986. **146**(7): p. 1309-11.

2. Aylett, M., et al., *Stopping drug treatment of hypertension: experience in 18 British general practices.* British Journal of General Practice, 1999. **49**(449): p. 977-80.

3. Beltman, F.W., et al., *Predictive value of ambulatory blood pressure shortly after withdrawal of antihypertensive drugs in primary care patients.* BMJ, 1996. **313**(7054): p. 404-6.

4. Danielson, M. and M. Lundback, *Withdrawal of antihypertensive drugs in mild hypertension.* Acta Medica Scandinavica - Supplementum, 1981. **646**: p. 127-31.

5. Ekbom, T., et al., *A 5-year prospective, observational study of the withdrawal of antihypertensive treatment in elderly people.* Journal of Internal Medicine, 1994. **235**(6): p. 581-8.

6. Fernandez, P.G., A.B. Galway, and B.K. Kim, *Prolonged normotension following cessation of therapy in uncomplicated essential hypertension.* Clinical & Investigative Medicine - Medecine Clinique et Experimentale, 1982. **5**(1): p. 31-7.

7. Fernandez, P.G., et al., *Separation of essential hypertensive patients based on blood pressure responses after the withdrawal of antihypertensive agents by step-wise discriminant analysis.* Current Medical Research and Opinion, 1983. **8**(7): p. 509-517.

8. Fotherby, M.D. and J.F. Potter, *Possibilities for antihypertensive drug therapy withdrawal in the elderly.* Journal of Human Hypertension, 1994. **8**(11): p. 857-63.

9. Freis, E.D., et al., *Effects of reduction in drugs or dosage after long-term control of systemic hypertension.* American Journal of Cardiology, 1989. **63**(11): p. 702-8.

10. Hansen, A.G., et al., *Withdrawal of antihypertensive drugs in the elderly.* Acta Medica Scandinavica - Supplementum, 1983. **676**: p. 178-85.

11. Herlitz, H., et al., *Different patterns of cellular sodium turnover in essential hypertension.* Journal of Hypertension - Supplement, 1988. **6**(4): p. S213-5.

12. Jennings, G.L., et al., *Factors influencing the success of withdrawal of antihypertensive drug therapy.* Blood Pressure Supplement, 1995. **2**: p. 99-107.

13. Langford, H.G., et al., *Return of hypertension after withdrawal of prolonged antihypertensive therapy, effect of weight loss, sodium reduction, and baseline factors.* Transactions of the Association of American Physicians, 1984. **97**: p. 190-6.

14. Lernfelt, B., et al., *Overtreatment of hypertension in the elderly?* Journal of Hypertension, 1990. **8**(5): p. 483-90.

15. Hypertension, M.R.C.W.P.o.M., *Course of blood pressure in mild hypertensives after withdrawal of long term antihypertensive treatment. Medical Research Council Working Party on Mild Hypertension.* British Medical Journal Clinical Research Ed., 1986. **293**(6553): p. 988-92.

16. Mitchell, A., et al., *The likelihood of remaining normotensive following antihypertensive drug withdrawal.* Journal of General Internal Medicine, 1989. **4**(3): p. 221-5.

17. Myers, M.G., et al., *Overtreatment of hypertension in the community?* American Journal of Hypertension, 1996. **9**(5): p. 419-25.

18. Nelson, M.R., et al., *Predictors of normotension on withdrawal of antihypertensive drugs in elderly patients: prospective study in second Australian national blood pressure study cohort.* BMJ, 2002. **325**(7368): p. 815.

19. Nelson, M.R., et al., *Short-term predictors of maintenance of normotension after withdrawal of antihypertensive drugs in the second Australian National Blood Pressure Study (ANBP2).* American Journal of Hypertension, 2003. **16**(1): p. 39-45.

20. Prasad, N., et al., *Safe withdrawal of monotherapy for hypertension is poorly effective and not likely to reduce health-care costs.* Journal of Hypertension, 1997. **15**(12 Pt 1): p. 1519-26.

21. Preston, R.A., et al., *Placebo-associated blood pressure response and adverse effects in the treatment of hypertension: observations from a Department of Veterans Affairs Cooperative Study.* Archives of Internal Medicine, 2000. **160**(10): p. 1449-54.

22. Sasamura, H., et al., *Feasibility of regression of hypertension using contemporary antihypertensive agents.* American Journal of Hypertension, 2013. **26**(12): p. 1381-1388.

23. Sieg-Dobrescu, D., et al., *The return of increased blood pressure after discontinuation of antihypertensive treatment is associated with an impaired post-ischemic skin blood flow response.* Journal of Hypertension, 2001. **19**(8): p. 1387-92.

24. Schmieder, R.E., et al., *Discontinuation of antihypertensive therapy: prevalence of relapses and predictors of successful withdrawal in a hypertensive community.* Cardiology, 1997. **88**(3): p. 277-84.

25. van den Bosch, W.J., et al., *Withdrawal of antihypertensive drugs in selected patients.* Lancet, 1994. **343**(8906): p. 1157.

26. van Duijn, H.J., et al., *Revised guidelines for cardiovascular risk management - time to stop medication? A practice-based intervention study.* British Journal of General Practice, 2011. **61**(587): p. e347-52.

27. Agents, V.A.C.S.G.o.A., *Return of elevated blood pressure after withdrawal of antihypertensive drugs.* Circulation, 1975. **51**(6): p. 1107-13.

28. Wahi, S., S. Sagar, and B.K. Sharma, *Is a hypertensive always a hypertensive?* Journal of the Association of Physicians of India, 1993. **41**(4): p. 198-9.

Supplementary digital content 2: Studies reporting adverse events or changes potentially leading to adverse events following withdrawal of AHT

| **Author Date Country** | **Study design** | **Threshold for hypertension in mmHg** | **Sample size and age** | **Eligibility criteria** | **Study outcomes** | **AHT used before withdrawal** | **Changes following withdrawal** | **Study limitations** |
| --- | --- | --- | --- | --- | --- | --- | --- | --- |
| Ames & Hill, 1982; USA [1] | Prospective cohort study with 4-21 weeks follow up | Dias BP>90 | n = 35; mean age = 53 (SD 9); 25 men, 10 women | Inclusion: on conventional AHT | Changes of glycohemoglobin and indices of lipid metabolism following withdrawal of AHT | Any | Total serum cholesterol sig. decreased after withdrawal of diuretics and combination therapy; triglyceride decreased sig after discontinuation of combination therapy but not diuretics; the ratio of total cholesterol to HDL cholesterol sig decreased after discontinuation of diuretics but not of combination therapy; glycohemoglobin was sig. lower after discontinuation of combination therapy but not of diuretics; one participant developed headache after withdrawal of chlorthalidone. | Small sample size; |
| Andersen et al., 2003; Denmark [2] | RCT for treatment of people with diabetes with irbesartan; 1 month withdrawal period after trial | 165/95 | n= 91 for those withdrawing after trial; placebo: n = 48; mean age = 57 (SD 9); men n = 35; irbesartan 150mg: n = 42; mean age = 57 (SD 9); men n = 33; irbesartan 300mg: n = 43; mean age = 55 (SD 9); men n = 31; | Inclusion: people with HT, type 2 diabetes and microalbuminuria | Change of mean BP levels after withdrawal of AHT; excretion rate of urinary albumin | Irbesartan | BP levels were unchanged at follow up in placebo group but had sig increased in irbesartan groups; following withdrawal, urinary albumin excretion rate did not change sig. in the placebo group or irbesartan 300mg group but sig increased in irbesartan 150mg group (by 68%); | Small sample sizes; |
| Andersen et al., 2008; Iceland (same study as Andersen et al., 2009, different outcomes reported) [3] | Withdrawal RCT following AHT RCT with 1 month withdrawal period after trial | - | Following re- randomisation before drug withdrawal: aliskiren withdrawal group: n= 163; aliskiren treatment group n = 170; ramipril withdrawal group n = 177; ramipril treatment group = 165; mean age: aliskiren group = 54.2 (SD 11.2); ramipril group = 53.0 (SD 10.1), 58.3% men in aliskiren group; 71% men in Ramipril group | Inclusion : age 18+; dias BP 90-110; exclusion: BP> 180/110; secondary hypertension; diabetes; history of cerebrovascular or cardiovascular disease; any condition that may affect the evaluation of efficacy or safety data or alter the absorption, distribution , metabolism or excretion of study drugs; | Efficacy, safety and tolerability of aliskiren and ramipril; | Aliskiren and ramipril | Following withdrawal, BP increased more rapidly in ramipril group compared to aliskiren group; incidents of adverse events during withdrawal were similar in those who stopped and continued aliskiren. Those who stopped ramipril or continued had higher rates of AE than those in the aliskiren group but there was no significant difference between continuing and stopping ramipril treatment; 3 SAEs occurred but none were considered related to study medication; changes in biochemistry and haematology parameters during withdrawal were small with no clinically meaningful differences between groups | Large sample size; same study as Anderson et al., 2009; |
| Andersen et al., 2009; Iceland [4] | Withdrawal RCT following AHT RCT with 1 month withdrawal period after trial | - | Following re- randomisation before drug withdrawal: aliskiren withdrawal group: n= 163; aliskiren treatment group n = 170; ramipril withdrawal group n = 177; ramipril treatment group = 165; mean age: aliskiren group = 54.2 (SD 11.2); ramipril group = 53.0 (SD 10.1), 58.3% men in aliskiren group; 71% men in Ramipril group | Inclusion : age 18+; dias BP 90-110; exclusion: BP> 180/110; secondary hypertension; diabetes; history of cerebrovascular or cardiovascular disease; any condition that may affect the evaluation of efficacy or safety data or alter the absorption, distribution , metabolism or excretion of study drugs; | Change in BP levels after withdrawal; plasma renin activity (PRA) and plasma renin concentration (PRC) | Aliskiren and ramipril | BP reductions persisted for longer after withdrawing aliskiren compared to ramipril; most increase occurred 1 week after stopping ramipril, while BP increased more gradually after stopping aliskiren; PRC decreased within 2 weeks after withdrawing AHT to pre-treatment levels; PRA levels were increased in those withdrawing from aliskiren but decreased rapidly in those who had stopped ramipril; geometric mean valuses for urinary albumin creatinine ratio did not change significantly | Large sample size; same study as Anderson et al., 2008; |
| Bishu et al., 2006; USA [5] | Prospective cohort study with a max of 5 week withdrawal period | 150/90 using ABPM | n = 41; mean age = 56.2 years (SD 14.1); men n = 19 (46%); | Inclusion: age 18+; diagnosed with end stage renal disease; treated with haemodialysis (HD) for at least 3 months and hypertension; exclusion: atrial fibrillation, BMI ≥ 40; history of missing one or more HD in the previous month; known drug abuse; severe chronic obstructive airway disease; stroke or myocardial infarction within the previous 6 months | Proportion of chronic HD patients remain normotensive when AHT was withdrawn | Any | Echocardiograms after the withdrawal period showed that the mean left ventricular mass index in those who remained normotensive was sig lower compared to those who remained hypertensive; sig less people who remained normotensive had left ventricular hypertrophy compared to those who were hypertensive at the end of the withdrawal period | Small sample size; |
| Bruce et al., 1979; USA [6] | Report of 2 case studies | n/a | n=2; age 54 and 52; both men; | Not reported | Effects of clonidine withdrawal | Clonidine | Rebound hypertension after 12h following abrupt withdrawal; | Case studies |
| Byyny et al., 1996; USA [7] | RCT followed by 24h withdrawal | n/a | n=100; mean age = 54.3 (SD9.8) years; 60 men | Inclusion: mild to moderate HT (dias BP 95-120 mmHg); age 21-72 years; within 30% of body weight; exclusion: black people; active medical problems that might affect the treatment of their HT; no drug treatment that might affect BP | Safety of Losartan | Losartan and Enalapril | BP returned slowly towards baseline in all treatment groups after 24 hours; no clinically important patterns in mean changes in pulse rate for any of the treatment group (not statistically tested) | Sample includes men only; short withdrawal follow-up period; |
| Caruana et al., 1985; UK [8] | Prospective cohort study with a 24 h withdrawal period following treatment with pinacidil | n/a | n=9; age and sex not reported; | Inclusion: patient at a hypertension clinic | Duration of action and useful dose-range of pinacidil | Pinacidil | No evidence of rebound HT | Very small sample size; demo-graphic character-istics not reported; short withdrawal follow-up period; |
| Cooper et al., 1988; UK [9] | Comparison of previously treated and untreated patients with HT following a 2 week withdrawal period | n/a | n =11710; age range: 18-97; 45.3% male; previously treated n= 7210 (61.6%); previously not treated n=4500 (38.4%) | Inclusion: diagnosed with HT | Effect of therapy withdrawal | Any | A sig increase was found in frequency of headache following withdrawal in all AHT medication groups, except in previously untreated patients and in those on a calcium antagonist | Very large sample size; type of statistical test not reported |
| De Cesaris et al., 1993; Italy [10] | 4 week prospective withdrawal study following a 10 months RCT comparing lisinopril and metoprolol | 150/90 | Total n = 44; lisinopril group n=22; mean age = 47.2 (SD 10); 11 men; metoprolol group n = 22; mean age = 46.1 (SD 9.4); 12 men; | Inclusion: mild to moderate essential hypertension as documented on three outpatient visits | Effects of lisinopril and metoprolol | Lisinopril and metoprolol | Lisinopril group: sig increase in brachial artery diameter and compliance and a decrease in vascular resistance and pulse wave velocity persisted after 4 weeks of withdrawal (values similar to those during treatment); Metoprolol group: no change in forearm hemodynamics; | Small sample size; |
| Ekbom et al., 1994; Sweden [11] | Observational study with a 5 year follow-up including participants of the wash-out phase of the STOP -Hypertension trial | No level set, restarting AHT depended on GP advice | n=333; mean age= 75.2 (SD 3.8; range 70-84); 32% men | Inclusion: hypertension; between 70 to 84 years old | Time until AHT was re-started | Any | No sig. difference between groups in total death hazard; those withdrawing had a sig. lower risk of cardiovascular events compared to those remaining in the treatment arms of the STOP-Hypertension trial | Very large sample size; sample |
| Eldridge et al., 1984; USA [12] | One week prospective withdrawal study following a 24 months RCT comparing hydrochlorothiazise (HCTZ) and guanabenz | n/a | Total n = 45; age range: 28 to 75; 20 men | Inclusion: essential HT; exclusion: clinically significant cardiac, hepatic, renal or endocrinologic impairments | Endocrinologic effects of HCTZ and Guanabenz | HCTZ and Guanabenz | No changes in levels of growth hormones, prolactin or insulin following withdrawal of HCTZ or guanabenz; no changes in glucagon levels following guanabenz withdrawal; glucagon levels sig decreased following HCTZ withdrawal; | Small sample size; |
| Fotherby & Potter, 1994a; UK [13] | Prospective cohort study with a 1 year follow up | 175/100 | n = 47; median age= 76 years, (range 68-82); | Inclusion: ≥ 65 years; BP <175/100 mmHg; on AHT for more than one year; currently attending hypertension clinic; exclusion: MI or stroke within the last 6 months; angina or known major illness, diabetes and other diseases to affect BP | Orthostatic HT | Thiazide diuretics, calcium antagonists and beta-adrenoceptor antagonists | Following withdrawal from AHT, numbers of people having orthostatic HT sig. fell from 11 (23%) to 4 (11%) by 12 months | Small sample size; participant characteristics not well reported; |
| Freis et al, 1989; USA [14] | RCT comparing AHT reduction, AHT withdrawal and control group after baseline phase of receiving different doses of hydrochlorothiazide (HCTZ) followed up for up to 30 months | Diastolic BP 85-90 depending on previously or newly diagnosed; no threshold for systolic BP | total n=606; AHT discontinued n = 137; mean age = 56.9 years; all men; | Inclusion: previously diagnosed with a dias BP was <115 mmHg and newly diagnosed patients with a dias BP between 90-95 mmHg; exclusion: malignant HT, haemorrhagic stroke, recent myocardial infarction, history of alcoholism or drug abuse, contraindication to study; | Number of participants who maintained control BP; changes in blood chemistry (glucose, potassium, cholesterol, triglycerides, uric acid and alkaline phosphatase) | HCTZ | No sig. changes in glucose and triglycerides levels; sig. increase in serum potassium and alkaline phosphatase levels and a sig. decrease in serum uric acid at 6 months in withdrawal group; | Very large sample size; only low dose HCTZ discontinued included men only; |
| Gerlis & Wright, 1980; UK [15] | Prospective cohort study of 12 months tiamenidine treatment with a 36 h follow-up after withdrawal of AHT | n/a | n = 10; mean age = 47 years; men n = 3; | Inclusion: essential HT; pre-treatment resting BP > 150/100 mmHg; | Effects of abrupt withdrawal; outcomes incl. rebound HT and biochemistries to assess catecholamine activity (urea, electrolytes, uric acid, haemoglobin, urinary sodium and metanephine levels) | Tiamenidine | Slight but non-sig. raise of pulse rate following withdrawal; no consistent evidence of excess catecholamine activity; evidence of systolic but not diastolic rebound HT following abrupt withdrawal; | Very small sample size; short withdrawal follow-up period; |
| Goldberg et al., 1977; UK [16] | Prospective cohort study with 3 day follow-up | n/a | n = 26; age range: 31 - 68; men n =15; | Inclusion: single drug AHT | Immediate effects of withdrawal on heart rate | Any | AHTs were categorised into 4 groups: clonidine, postganglionic neurone-blocking drugs, methyldopa and beta blockers; clonidine withdrawal: sig. increase in heart rate with intense ectopic activity; postganglionic neurone-blocking drug withdrawal: similar but less pronounced reaction with increased ventricular ectopic activity; no alarming reactions after withdrawal of metyldopa or beta blockers; | Small sample size; short withdrawal follow-up period; |
| Hajjar et al, 2013; US [17] | Prospective cohort study with a 3-4 week follow-up | n/a | n = 53; mean age = 71 (SD 7) years; 44% men; | Inclusion: currently receiving AHT | Safety of short term AHT withdrawal | Any | In 2% of readings BP exceeded 180/100 mmHg but not in 2 consecutive readings; none of the participants reported headaches, dizziness, visual changes or focal weakness during withdrawal phase; | Small sample size; |
| Hansen et al., 1995 Denmark [18] | Prospective cohort study with a 1 month follow-up | n/a | n = 42; mean age = 40 (SD 7); men n = 26; | Inclusion: patients who are on AHT and insulin dependent with diabetic nephropathy | BP, GFR and albuminuria | Any | BP, GFR and albuminuria were all sig increased after 1 month withdrawal(although 20-25% of participants had unchanged BP, GFR and or albuminuria) | Small sample size; |
| Hansen et al., 1997; Denmark [19] | Prospective cohort study with a 1 month follow-up | n/a | n = 40; mean age = 61 (SD 7); men n = 29; | Inclusion: GFR> 25 ml · min^-1^ · 1.73m^-2^; receiving AHT for > 1 year; age between 18 and 75 years; not insulin dependent | BP, GFR and albuminuria | Any | BP and albuminuria levels sig increased after 4 weeks of withdrawal. There was no sig change in GFR. 10-17% of participants had a decrease in BP and/or albuminuria | Small sample size; |
| Hansson & Hökfelt, 1981; Sweden [20] | Withdrawal phases (3 days) in cross-over study assessing effects of tiamenidine or clonidine | n/a | Sample size and age not reported; all men; | Inclusion: primary essential moderate HT, between 20 and 65 years old, male; exclusion: concomitant renal, cardiovascular or cerebrovascular complications or severe intercurrent illness, receiving sedatives, tranquillisers, antidepressants, cardiac glycosides or diuretics | BP, weight change, plasma noradrenaline, plasma adrenaline, urinary catecholamines and plasma renin activity | Tiamenidine or clonidine | BP, plasma noradrenaline, plasma adrenaline, urinary catecholamines and plasma renin activity levels after withdrawal overshot baseline levels; no sig. change in weight; | Sample size and demographics not reported; only men included; short withdrawal follow-up period; |
| Ikeda et al., 1997; USA; [21] | One week cross-sectional withdrawal study following 12 weeks of AHT RCT | n/a | n = 366; mean age 54 (range 26-78); 64% men | Inclusion: mild to moderate HT, at least 21 years old; exclusion: no medical problems that might affect treatment of HT, no drugs that might affect BP, pregnant or lactating females; medical conditions that might put patient at undue risk, adversely affect participation in trial or compromise quality of data obtained | Efficacy and safety of AHT | Losartan | No evidence of rebound HT | Large sample size; |
| Jennings et al., 1984; Australia [22] | Prospective cohort study with a up to 20 week follow-up | n/a | n = 11; mean age = 51.5 (SD 3.4); men n = 5; | Inclusion: Uncomplicated essential hypertension which has been treated for at least 1 year; BP < 160/90 mmHg at last visit | BP levels after withdrawal, total peripheral resistance index (TPRI) , plasma renin activity, noradrenaline and adrenaline, 24h urinary aldosterone and electrolyte excretion | Any | Following withdrawal the non-autonomic TPRI raised sig. to a normal level; plasma renin activity rose following withdrawal of diuretics but remained constant thereafter; noradrenaline, adrenaline, 24h urinary aldosterone and electrolyte levels as expected after drug withdrawal; | Very small sample size; |
| Kirch & Distler, 1978; Germany [23] | Double blind crossover study with 2 week withdrawal period | n/a | n = 21; mean age = 48.5 years (range 18-63); men n = 12; | Inclusion: essential HT; exclusion: any form of secondary HT; | Effectiveness and side effects of N- amidino-2- acetamide hydrochloride and clonidine | N-amidino-2- acetamide hydrochloride and clonidine | Two days after abruptly stopping AHT rebound HT was determined following clonidine treatment only | Small sample size; recruitment and selection not well reported; |
| Lacourciere et al., 1998; Canada [24] | AHT RCT followed by a 2 week wash-out period | n/a | n = 278; mean age = 53.4 (SD 9.5) years; 67% men; | Inclusion: mild to moderate HT; age between 21 and 70 years; exclusion: secondary HT; cardiac, pulmonary, renal, neurologic, hepatic, hematologic or metabolic disease, hypersensitivity to angiotensin II antagonists or using drugs acting on BP; women with childbearing potential | Effects of 4 doses of tasosartan | Tasosartan | No rebound HT after withdrawal of AHT | Very large sample size; well reported study; |
| Lernfelt et al; 1990; Sweden [25] | Prospective cohort study with 2 year follow-up | 200/105 | n=25; age = 70; 10 men (40%) | Inclusion: part of the '70 year old people in Gothenburg, Sweden' study; using AHT; exclusion: BP> 175/95 mmHg; cardiovascular disease; EEG abnormalities; increased heart volume; other serious diseases | BP, heart rate, left ventricular (LV) morphology, LV diastolic function, LV ejection phase indices, LV wall stress, ECG | Any | No sig. change in LV morphology and LV diastolic function; sig. decrease in fractional shortening and ejection fraction; slight but sig. increase in end-systolic wall stress; four participants showed signs of cardiovascular disorders after withdrawal but the authors were unable to determine if this was a consequence of the withdrawal study; | Small sample size; |
| Ljungman et al., 1988; Sweden [26] | Cross sectional cohort study with 7 years of AHT and subsequent withdrawal | 170/105 | n = 120; mean age = 49; all men | Inclusion: 49 years old, male; exclusion: previous AHT, malignant or secondary HT; | Renal function (GFR, renal plasma flow, renal blood flow, renal vascular resistance) | Metoprolol, hydrochlorothiazide and hydralazine | BP levels returned to HT after a median time of 10 weeks; no sig changes in renal function after AHT withdrawal except for increase in renal vascular resistance | Large sample size; only men |
| Middeke et al., 1990; Germany [27] | RCT with subsequent withdrawal study for 4 weeks | n/a | n = 40; age not reported; all male | Inclusion: essential hypertension | Cholesterol, high and low density lipoproteins, triglycerides in serum | Diuretics: hydrochlorothiazide (in n = 23) and beta blocker: atenolol ( in n = 17) | No sig change in heart rate in diuretic group but sig increase in heart rate in beta blocker group following withdrawal; after discontinuation of diuretics sig decrease in total cholesterol and low density lipoproteins, but no sig change in high density lipoproteins or triglycerides; following withdrawal of beta blockers sig decrease of low density lipoproteins and triglycerides but sig increase of high density lipoproteins | Small sample size; recruitment and selection not well reported; |
| Miller et al., 1994; USA [28] | Open label trial with quinapril followed by a 4 week withdrawal RCT including a placebo and a quinapril treatment groups for moderate renal impairment and chronic renal failure | n/a | Moderate renal impairment (MRI) group: placebo: n=10; mean age= 42 (SD 3.5); men n = 5; quinapril: n = 10; mean age= 48 (SD 3.6); men n= 8; chronic renal failure (CRF) group: placebo: n=13; mean age = 55 (SD 3.7); men n= 10; quinapril: n= 15; mean age = 53 (SD 3.5), men n = 5; | Inclusion: moderate renal impairment or chronic renal failure | BP levels after withdrawal of AHT; change in creatinine clearance; mean urinary protein; blood urea nitrogen | Quinapril | Sig increase of mean sitting BP in placebo compared to quinapril groups (MRI and CRF); no sig difference in creatinine clearance between groups; sig greater increase of mean urinary protein in placebo compared to quinapril group in patients with CRF but in patients with MRI only borderline sig; mean blood urea nitrogen decreased sig in placebo compared with quinapil group in CRF group but not in MRI group; | Very small sample sizes; |
| Muiesan et al., 2000; Italy [29] | Prospective cohort study with treatment and 4 week withdrawal phase | n/a | n = 68; mean age = 47 (SD 9) years; age range: 22-62; men n = 50; | Inclusion: uncomplicated HT, prior to study untreated; exclusion: coronary and/or vascular heart disease, heart failure | Changes in left ventricular (LV) performance | Angiotensin converting enzyme inhibitors, calcium antagonists or a combination of both | No sig changes in LV mass index, relative wall thickness, endocardial or midwall fractional shortening and LV diastolic diameter following withdrawal; | Small sample size; |
| Myers et al., 1996; Canada [30] | Cohort withdrawal study with regular BP monitoring at 1,2,4,8 weeks and then 6-8 weeks intervals (incl. ambulatory monitoring) | Mean of two ambulatory BP recordings exceeding 160/95 for restart of medication; 150/90 and 140/90 cut-offs were also considered for % remaining normotensive at 1 year | n=98; mean age: 62 years (SD 2; range 21-80); men n = 36; | Inclusion: receiving AHT exclusion: target organ damage, secondary HT, cardiac arrhythmias, receiving other medication affecting their BP; if mean ambulatory sys BP ≥160 or dias BP ≥ 95 mmHg; if AHT had been changed in the last 3 months | Proportion of patients remaining off treatment at 1 year | Any | Withdrawal of AHT had not effect on left ventricular mass |  |
| Nami et al., 1983; Italy [31] | AHT RCT followed by a 6 day withdrawal period | n/a | Clonidine group: n = 10; mean age = 60.8 (SD 914); men n= 5; | Inclusion: stable mild to moderate HT who had not been refractory to previous single drug therapy; supine dias BP 95-120 mmHg after placebo run-in period exclusion: target organ damage, diabetes, liver or renal diseases; pregnant women, people with alcohol problems; | BP, heart rate, urinary catecholamines, cyclic nuceotides | Clonidine or guanfacine (not reported as out-dated) | Clonidine group: BP levels and pulse rate returned to pre-treatment levels within 2 days and sometimes went sig. above them in the following days (no stat. test results reported); Urinary catecholamine and urinary cyclic nucleotides levels returned to baseline values and to higher (but not sig higher) levels the following days; side effects of withdrawal of clonidine included headache ( in 9 out of 10 pp), palpitations (5/10), dizziness (2/10), sweating (2/10) and facial flushing (1/10); | Very small sample size; recruitment not reported; short withdrawal follow-up period; |
| Panza et al., 1993; USA [32] | Case-control AHT study with a 2 weeks withdrawal period | n/a | HT group: n = 15; mean age = 54.1 (SD 12) years; men n = 11; control group (without history of HT) n= 15; mean age = 52.3 (SD 7) years; men n= 10 | Inclusion HT group: history of chronically elevated BP (≥145/95 mmHg); no apparent underlying cause of HT; normotensive when on AHT; exclusion: BP remained elevated despite AHT; control group: matched cases without HT | Response of vasculature following withdrawal | Any | During withdrawal, increase in blood flow and decrease in vascular resistance with acetylcholine were sig lower in withdrawal group compared to control group (no history of HT); no sig differences between forearm blood flow and vascular resistance response to sodium nitroprusside between groups during withdrawal; heart rate was similar during treatment and withdrawal phases | Very small sample size; well reported study; |
| Pedersen, 1976; Denmark [33] | Crossover comparison of metoprolol and hydrocholothiazide with a 8 week placebo period between the two treatments | n/a | n = 20; mean age = 44 (range 29-63 years); men n = 10; | Inclusion: mild arterial essential HT; exclusion: chronic bronchitis, bronchial asthma, cardiac failure, diabetes mellitus | BP level and heart rate | Metoprolol and hydrochlorothiazide | Five out of 20 patients had subjective symptoms following withdrawal of metoprolol: general malaise, palpitations and headache | Small sample size; recruitment not reported; |
| Pedrinelli et al., 2000; Italy [34] | Two week cohort withdrawal study following AHT RCT | n/a | n = 28; age range: 33-77 years; all men | Inclusion: mild essential HT; exclusion: secondary HT | Skin blood flow (laser doppler flowmetry), leg weight | Amlodipine, enalapril, and enalapril and amlodipine combined | Resting and dependent skin flow as well as leg weight increased during treatment with amlodipine and amlodipine and enalapril combination (not enalapril alone) and recovered during withdrawal; | Small sample size; study includes men only; |
| Perlini et al., 2001; Italy [35] | One month cohort withdrawal study following AHT RCT | n/a | n = 152; age and sex not reported; | Inclusion: essential HT, age range 20-65 years, dias BP between 95-115 after 4 weeks without AHT, left ventricular hypertrophy (LVH); exclusion: history or signs of cardiovascular complications or major target organ damage, major cardiovascular or non- cardiovascular disease besides HT, pregnancy, lactation, contraindications to study drugs, conditions that would prevent data collection, atrial fibrillation, using >2 AHT | LVH | Lisinopril or lininopril and hydrochlorothiazide | During withdrawal after AHT RCT, only one third of treatment induced LVH regression was reversed; Circumferential end-systolic stress returned to pre-treatment values after AHT withdrawal; improvement in midwall fractional shortening persisted after withdrawal period; BP levels returned to pre-treatment levels following withdrawal; relative wall thickness was reduced by AHT and did not change after withdrawal; | Large sample size; demographic characteristics not reported; |
| Petretta et al., 1996; Italy [36] | Prospective cohort study with 1 month withdrawal | n/a | n = 30; mean age = 48 (SD 8); men n= 18 | Inclusion: uncomplicated essential HT, LVH; Exclusion: age > 70 years, diagnosis of angina, myocardial infarction, vascular heart disease, heart failure, atrial fibrillation, diabetes mellitus, renal failure, obesity, poor exercise capacity as a result of a lung disease, musculoskeletal problems, decreased left ventricular systolic function , abnormal or doubtful thallium stress tests results | Cardiac autonomic control | Lisinopril | Following withdrawal, BP rose to pre-treatment baseline levels; left ventricular mass index did not change after withdrawal; the increase of night-time high frequency power of heart period variability due to treatment remained detectable after withdrawal; in people with LVM normalisation, day and night-time high-frequency powers increased; in people with LVM reduction without normalisation power spectral measures of heart period variability were sig lower than in those with LVM normalisation. | Small sample size; recruitment not reported; |
| Plänitz, 1984; Germany [37] | Three day withdrawal study following 4 weeks of a AHT RCT cross-over study | n/a | Moxonidine followed by clonidine group: n = 10; mean age= 41 (SD 5.8) years; men n= 7; clonidine followed by moxonidine group: n = 10; mean age 39 (SD 7.5) years, men n = 6 | Inclusion: uncomplicated essential HT; exclusion: additional ailments | BP levels and withdrawal reactions | Moxonidine and clonidine | After abrupt discontinuation of clonidine therapy, there was a more rapid rise in BP than after stop of moxonidine therapy; There was no indication of overshoot in either treatment group; no subjective withdrawal symptoms such as anxiety, insomnia, sweating, headache or tachycardia were observed after abrupt stop of either drug. | Very small sample size; short withdrawal follow-up period |
| Pool et al., 1998; USA [38] | One week withdrawal study following 8 weeks of AHT RCT | n/a | n = 570; mean age = 54.2 years (SD 10.3); 67% men; | Inclusion: age 18 years or older; well established history of mild to moderate HT, women sterile or post- menopausal; exclusion: diseases that would present safety hazards or concomitant medications that might interfere with the assessment of the safety or efficacy of irbesartan | Efficacy, safety, pharmacokinetics and pharmacodynamics | Irbesartan | No indication of rebound HT | Very large sample size; |
| Prasad et al, 1997; UK [39] | Prospective cohort study with 1 year follow-up | Daytime ABPM BP > 150/90 | n= 36; median age = 64 (range 51-82); 17 women, 19 men | Inclusion: on antihypertensive monotherapy; exclusion: ischaemic heart disease, cerebrovascular disease, peripheral vascular disease, diabetes, renal impairment, left ventricular hypertrophy, secondary HT; | Proportion of people who remained normotensive at 52 weeks after withdrawal | Any | No changes in left ventricular mass and left ventricular dias filling parameters following withdrawal | Small sample size; |
| Preston et al., 2000; USA [40] | RCT including active treatment and placebo groups with 1 year follow-up of the placebo group | Dias BP>95 | n = 1292; active treatment group: n = 1105; placebo group n = 187; no mean age reported; all men; | Inclusion: age > 21 years, reasonable expectation that dias BP would be between 95 and 105 mmHg with placebo; exclusion: secondary HT | Response to placebo at 1 year follow up and effect of race and age on response | Not reported | Headache and joint pain was sig more often reported in the placebo group compared to the treatment group and sleepiness was sig more often reported in the active treatment group compared to the placebo group. There was no difference in fatigue, weakness, nightmares, vivid dreams, sleep disturbance, stomach cramps, diarrhoea, constipation, vertigo, dizziness, nausea, muscle cramps, impotence, loss of libido, cold extremities, palpitations, chest pain, change in taste or dry mouth | Very large sample size; sample not well described;; |
| Saul et al., 2013; USA [41] | One month withdrawal study following AHT RCT | n/a | Carvedilol and lisinopril group: n = 23, mean age = 52 (SD 13) years, men n = 17; Carvedilol group n = 24, age = 53 (SD 10) years, men n = 17; Lisinopril group n = 21, age = 53 (SD 10) years, men n = 16; Placebo group n = 23, age = 51 (SD 13) years, men n = 15; | BP prehypertensive or hypertensive (≥ 130/85 mmHg), not taking AHT, additional risk factor; exclusion: previous cardiovascular events, current pregnancy or lactation, use of AHT or other cardiac medication, other serious medical conditions; | Cardiovascular health | Carvedilol, lisinopril and a combination of both | All changes due to AHT were reversed following withdrawal (sig decrease of BP levels, small borderline sig improvement of disease score for cardiovascular health (scores for small and large artery elasticity, resting BP, exercise BP, retinal analysis, electrocardiography, carotid intimal media thickness, LVM, microalbuminuria, N-terminal pro B-type natriuretic peptide) and metabolic parameters; | Small sample size; sig. of changes of individual parameters not reported; |
| Schmieder et al., 1985; Germany [42] | Prospective cohort study with a 5 months follow up | 159/94 | n= 47; mean age= 43.5 (SD 6 years); all men | Inclusion: essential hypertension, AHT monotherapy for 6 months | BP levels after withdrawal | Oxprenolol and nitrendipine | Sig higher standing dias BP levels in people withdrawn from nitrendipine compared to those withdrawn from oxprenolol; at 2 weeks sig higher heart rate in erect position in those previously on oxprenolol compared to those on nitrendipine; at later measurements these differences disappeared | Small sample size; study includes men only; |
| Schneider et al., 2012; Germany [43] | Four to five days withdrawal study following AHT RCT | n/a | n = 34; mean age = 54 years (SD 9; range 28-71); men n= 27 | Inclusion: mean BP > 140/90 mmHg or treated arterial HT; exclusion: clinically sig liver or kidney disease, atrial fibrillation, atrioventricular blockade grade II or higher and current treatment with aliskiren | Renal perfusion (measured by MRI) | Aliskiren | Following withdrawal, sys and dias BP levels did not change; increase in renal perfusion, and decrease in angiotensin II and aldosterone levels due to Aliskiren was reversed following withdrawal; no changes following withdrawal in urinary angiotensinogen levels; plasma renin activity remained reduced following withdrawal; | Small sample size; short follow-up; recruitment not reported; |
| Sieg-Dobrescu et al., 2001; Switzerland [44] | Withdrawal RCT with 24 week follow-up | 140/90 | Discontinuation group: mean n = 20; mean age = 48 years (SD 14.3); men n = 10; treatment continuation group: n = 20; mean age = 47 years (SD 13.2); men n = 11 | Inclusion: receiving AHT for at least previous 6 months, BP < 140/90 mmHg; exclusion: requiring treatment for hypercholesterolemia, diabetes, angina pectoris or congestive heart failure, previous cardiovascular event | BP levels after withdrawal | Any | Post-ischemic skin blood flow responses decreased sig in those who were successfully withdrawn from AHT compared to those continuing or re-starting AHT who showed no change in blood flow responses; | Very small sample size; |
| Smith et al., 1986; UK [45] | Cohort treatment and withdrawal study with 24h follow-up | n/a | n = 9; mean age 46.3 years; men n = 5; | Inclusion: dias BP> 100; exclusion: organ target damage, malignant HT; | BP levels, heart rate, plasma renin activity and weight and ankle circumference after withdrawal | Felodipine | Heart rate fell sig below treatment level following withdrawal; sinoaortic baroreceptor heart rate (SAB-HR), plasma renin activity and weight and ankle circumference did not change sig; | Very small sample size; very short withdrawal period; |
| Trimarco et al., 1994; Italy [46] | One month withdrawal study following AHT cohort study | n/a | n = 11; mean age = 49 years (SD 1.6); men n = 9; | Inclusion: mild to moderate essential HT, left ventricular hypertrophy; exclusion: coronary artery disease, diabetes mellitus, renal insufficiency, heart failure, prior myocardial infarction, secondary HT; | Cardiovascular function and structure | Oxazoline (rilmenidine) | No sig differences between treatment and withdrawal period were found in left ventricular mass index, interventricular septum and posterior wall thickness; improvements of left ventricular diastolic function observed during treatment returned to baseline following washout; effects on forearm circulation (pulse wave velocity) and brachial artery compliance did not change after withdrawal of oxazoline; reduction in forearm vascular resistance during AHT returned to baseline after withdrawal; plasma atrial natriuretic factor levels sig decreased following AHT withdrawal | Very small sample size; |
| Van Duijn et al., 2011; Netherlands [47] | Prospective cohort study with 6 months follow up | Low cardiovascular risk and patient's decision to stop or re-start AHT | n= 135; age: 25-75 years; | Inclusion: no known target organ damage, receiving AHT and/or medication for hypercholesterolaemia; exclusion: cardiovascular disease, taking drugs for other indications than HT or hypercholeserolaemia; | Attendance for re-evaluation | Any | Reasons for re-starting medication were headaches (n=12), ankle oedema (n=2), not feeling well n=9), high BP and/or high cholesterol (n=32). However, in 21 of 32 patients who restarted medication because of high BP or high cholesterol the cardiovascular mortality risk would not have been high enough to advise restarting medication according to Dutch guidelines; | Reasons to re-start medication reported in combination with withdrawal of medication for hypercholesterolaemia |
| Veterans Administration Cooperative Study Group on Antihypertensive Agents, 1975; USA [48] | RCT including continued treatment and withdrawal arm with a 72 week follow up | Dias BP > 129 at one visit or dias BP > 114 at any 2 visits or dias BP > 104 at any 3 visits or dias BP > 94 at any 5 visits or any major cardiovascular complications | Withdrawal group: n = 60 ; mean age = 52.2 years; treated group: n=26; mean age 52.8 years; all men | Inclusion: participant of previous AHT trial; dias BP for 2 preceding visits ≤ 90 mmHg; no dias BP > 95 mmHg during any of the 3 preceding visits; average dias BP during preceding 12 months ≤95 mmHg | Number of participants remaining below threshold BP | Hydrochlorothiazide, reserpine and hydralazine | Serum uric acid fell sig in withdrawal but not treatment group; serum potassium rose sig in withdrawal but not treatment group; Serum creatinine did not change sig in either group; | Small sample size; high dias BP threshold value; no sys BP threshold value set; |
| Wahi et al., 1993; India [49] | Prospective cohort study with a 1 year follow up | 160/90 at 2 readings or 200/110 at one reading | n = 42; mean age = 40.7; (range 22 to 65); 30 men; | Inclusion: 15-65 years; no secondary HT; on AHT; had BP on 2 occasions before start of AHT; at least 6 months of AHT; no contraindication to AHT withdrawal such as angina, cardiac failure, renal insufficiency or stroke; possibility to follow up for a minimum of 1 year; BP averaging less than 140/85mmHg in 6 months before withdrawal and 140/90 mmHg on start of withdrawal | Number of participants remaining below threshold BP | Any | No deterioration of biochemical profile (tests not reported) or renal function was observed in any of the participants | Small sample size; sample not well described; high BP threshold values; |

AHT: antihypertensive treatment; dias: diastolic; HT: hypertension; SAE: serious adverse event; sys: systolic; sig: significant;

1 Ames RP, Hill P. Improvement of glucose tolerance and lowering of glycohemoglobin and serum lipid concentrations after discontinuation of antihypertensive drug therapy. *Circulation* 1982; **65:** 899-904.

2 Andersen S, Brochner-Mortensen J, Parving HH, Irbesartan in Patients With Type D, Microalbuminuria Study G. Kidney function during and after withdrawal of long-term irbesartan treatment in patients with type 2 diabetes and microalbuminuria. *Diabetes Care* 2003; **26:** 3296-302.

3 Andersen K, Weinberger MH, Egan B, Constance CM, Ali MA, Jin J, Keefe DL. Comparative efficacy and safety of aliskiren, an oral direct renin inhibitor, and ramipril in hypertension: a 6-month, randomized, double-blind trial. *J Hypertens* 2008; **26:** 589-99.

4 Andersen K, Weinberger MH, Constance CM, Ali MA, Jin J, Prescott MF, Keefe DL. Comparative effects of aliskiren-based and ramipril-based therapy on the renin system during long-term (6 months) treatment and withdrawal in patients with hypertension. *J Renin Angiotensin Aldosterone Syst* 2009; **10:** 157-67.

5 Bishu K, Gricz KM, Chewaka S, Agarwal R. Appropriateness of antihypertensive drug therapy in hemodialysis patients. *Clin J Am Soc Nephrol* 2006; **1:** 820-4.

6 Bruce DL, Croley TF, Lee JS. Preoperative clonidine withdrawal syndrome. *Anesthesiology* 1979; **51:** 90-2.

7 Byyny RL, Merrill DD, Bradstreet TE, Sweet CS. An inpatient trial of the safety and efficacy of losartan compared with placebo and enalapril in patients with essential hypertension. *Cardiovasc Drugs Ther* 1996; **10:** 313-9.

8 Caruana MP, Al-Khawaja I, Royston P, Raftery EB. The effects of long-acting pinacidil on intra-arterial blood pressure. *Br J Clin Pharmacol* 1985; **20:** 140-3.

9 Cooper WD, Glover DR, Hormbrey JM. Symptoms in hypertensive patients: the effect of treatment withdrawal. *J Hypertens Suppl* 1988; **6:** S629-30.

10 De Cesaris R, Ranieri G, Filitti V, Andriani A, Bonfantino MV. Forearm arterial distensibility in patients with hypertension: comparative effects of long-term ACE inhibition and beta-blocking. *Clin Pharmacol Ther* 1993; **53:** 360-7.

11 Ekbom T, Lindholm LH, Oden A, Dahlof B, Hansson L, Wester PO, Schersten B. A 5-year prospective, observational study of the withdrawal of antihypertensive treatment in elderly people. *J Intern Med* 1994; **235:** 581-8.

12 Eldridge JC, Strandhoy J, Buckalew VM, Jr. Endocrinologic effects of antihypertensive therapy with guanabenz or hydrochlorothiazide. *J Cardiovasc Pharmacol* 1984; **6 Suppl 5:** S776-80.

13 Fotherby MD, Potter JF. Orthostatic hypotension and anti-hypertensive therapy in the elderly. *Postgrad Med J* 1994; **70:** 878-81.

14 Freis ED, Thomas JR, Fisher SG*, et al.* Effects of reduction in drugs or dosage after long-term control of systemic hypertension. *Am J Cardiol* 1989; **63:** 702-8.

15 Gerlis LS, Wright N. Abrupt withdrawal of the antihypertensive agent tiamenidine after 12-months' treatment. *Pharmatherapeutica* 1980; **2:** 408-11.

16 Goldberg AD, Raftery EB, Wilkinson P. Blood pressure and heart rate and withdrawal of antihypertensive drugs. *British Medical Journal* 1977; **1:** 1243-6.

17 Hajjar I, Hart M, Wan SH, Novak V. Safety and blood pressure trajectory of short-term withdrawal of antihypertensive medications in older adults: experience from a clinical trial sample. *J Am Soc Hypertens* 2013; **7:** 289-93.

18 Hansen HP, Rossing P, Tarnow L, Nielsen FS, Jensen BR, Parving HH. Increased glomerular filtration rate after withdrawal of long-term antihypertensive treatment in diabetic nephropathy. *Kidney Int* 1995; **47:** 1726-31.

19 Hansen HP, Nielsen FS, Rossing P, Jacobsen P, Jensen BR, Parving HH. Kidney function after withdrawal of long-term antihypertensive treatment in diabetic nephropathy. *Kidney Int Suppl* 1997; **63:** S49-53.

20 Hansson BG, Hokfelt B. Changes in blood pressure, plasma catecholamines and plasma renin activity during and after treatment with tiamenidine and clonidine. *Br J Clin Pharmacol* 1981; **11:** 73-7.

21 Ikeda LS, Harm SC, Arcuri KE, Goldberg AI, Sweet CS. Comparative antihypertensive effects of losartan 50 mg and losartan 50 mg titrated to 100 mg in patients with essential hypertension. *Blood Press* 1997; **6:** 35-43.

22 Jennings GL, Korner PI, Laufer E, Esler MD, Burton D, Bruce A. How hypertension redevelops after cessation of long-term therapy. *J Hypertens Suppl* 1984; **2:** S217-9.

23 Kirch W, Distler A. Antihypertensive effect of N-amidino-2-(2,6-dichlorophenyl) acetamide hydrochloride. A double-blind cross-over trial versus clonidine. *Int J Clin Pharmacol Biopharm* 1978; **16:** 132-5.

24 Lacourciere Y, Pool JL, Svetkey L, Gradman AH, Larochelle P, de Champlain J, Smith WB. A randomized, double-blind, placebo-controlled, parallel-group, multicenter trial of four doses of tasosartan in patients with essential hypertension. Tasosartan Investigator's Group. *Am J Hypertens* 1998; **11:** 454-61.

25 Lernfelt B, Landahl S, Svanborg A, Wikstrand J. Overtreatment of hypertension in the elderly? *J Hypertens* 1990; **8:** 483-90.

26 Ljungman S, Aurell M, Hartford M, Wikstrand J, Berglund G. Renal function before and after withdrawal of long term antihypertensive treatment in primary hypertension. *Drugs* 1988; **35 Suppl 5:** 55-8.

27 Middeke M, Richter WO, Schwandt P, Beck B, Holzgreve H. Normalization of lipid metabolism after withdrawal from antihypertensive long-term therapy with beta blockers and diuretics. *Arteriosclerosis* 1990; **10:** 145-7.

28 Miller MA, Texter M, Gmerek A, Robbins J, Shurzinske L, Canter D. Quinapril hydrochloride effects on renal function in patients with renal dysfunction and hypertension: a drug-withdrawal study. *Cardiovasc Drugs Ther* 1994; **8:** 271-5.

29 Muiesan ML, Salvetti M, Monteduro C*, et al.* Changes in midwall systolic performance and cardiac hypertrophy reduction in hypertensive patients. *J Hypertens* 2000; **18:** 1651-6.

30 Myers MG, Reeves RA, Oh PI, Joyner CD. Overtreatment of hypertension in the community? *Am J Hypertens* 1996; **9:** 419-25.

31 Nami R, Bianchini C, Fiorella G, Chierichetti SM, Gennari C. Comparison of effects of guanfacine and clonidine on blood pressure, heart rate, urinary catecholamines, and cyclic nucleotides during and after administration to patients with mild to moderate hypertension. *J Cardiovasc Pharmacol* 1983; **5:** 546-51.

32 Panza JA, Quyyumi AA, Callahan TS, Epstein SE. Effect of antihypertensive treatment on endothelium-dependent vascular relaxation in patients with essential hypertension. *J Am Coll Cardiol* 1993; **21:** 1145-51.

33 Pedersen OL. Comparison of metoprolol as hydrochlorothiazide and antihypertensive agents. *Eur J Clin Pharmacol* 1976; **10:** 381-5.

34 Pedrinelli R, Dell'Omo G, Melillo E, Mariani M. Amlodipine, enalapril, and dependent leg edema in essential hypertension. *Hypertension* 2000; **35:** 621-5.

35 Perlini S, Muiesan ML, Cuspidi C*, et al.* Midwall mechanics are improved after regression of hypertensive left ventricular hypertrophy and normalization of chamber geometry. *Circulation* 2001; **103:** 678-83.

36 Petretta M, Bonaduce D, Marciano F*, et al.* Effect of 1 year of lisinopril treatment on cardiac autonomic control in hypertensive patients with left ventricular hypertrophy. *Hypertension* 1996; **27:** 330-8.

37 Planitz V. Crossover comparison of moxonidine and clonidine in mild to moderate hypertension. *Eur J Clin Pharmacol* 1984; **27:** 147-52.

38 Pool JL, Guthrie RM, Littlejohn TW, 3rd*, et al.* Dose-related antihypertensive effects of irbesartan in patients with mild-to-moderate hypertension. *Am J Hypertens* 1998; **11:** 462-70.

39 Prasad N, Davey PG, Watson AD, Peebles L, MacDonald TM. Safe withdrawal of monotherapy for hypertension is poorly effective and not likely to reduce health-care costs. *J Hypertens* 1997; **15:** 1519-26.

40 Preston RA, Materson BJ, Reda DJ, Williams DW. Placebo-associated blood pressure response and adverse effects in the treatment of hypertension: observations from a Department of Veterans Affairs Cooperative Study. *Arch Intern Med* 2000; **160:** 1449-54.

41 Saul SM, Duprez DA, Zhong W, Grandits GA, Cohn JN. Effect of carvedilol, lisinopril and their combination on vascular and cardiac health in patients with borderline blood pressure: the DETECT Study. *J Hum Hypertens* 2013; **27:** 362-7.

42 Schmieder RE, Ruddel H, Neus H, Neus J, von Eiff AW. Predictors of blood pressure increases after withdrawal of antihypertensive therapy. *J Hypertens Suppl* 1985; **3:** S457-9.

43 Schneider MP, Janka R, Ziegler T*, et al.* Reversibility of the effects of aliskiren in the renal versus systemic circulation. *Clin J Am Soc Nephrol* 2012; **7:** 258-64.

44 Sieg-Dobrescu D, Burnier M, Hayoz D, Brunner HR, Waeber B. The return of increased blood pressure after discontinuation of antihypertensive treatment is associated with an impaired post-ischemic skin blood flow response. *J Hypertens* 2001; **19:** 1387-92.

45 Smith SA, Mace PJ, Littler WA. Felodipine, blood pressure, and cardiovascular reflexes in hypertensive humans. *Hypertension* 1986; **8:** 1172-8.

46 Trimarco B, Rosiello G, Sarno D, Lorino G, Rubattu S, DeLuca N, Volpe M. Effects of one-year treatment with rilmenidine on systemic hypertension-induced left ventricular hypertrophy in hypertensive patients. *Am J Cardiol* 1994; **74:** 36A-42A.

47 van Duijn HJ, Belo JN, Blom JW, Velberg ID, Assendelft WJ. Revised guidelines for cardiovascular risk management - time to stop medication? A practice-based intervention study. *Br J Gen Pract* 2011; **61:** e347-52.

48 Agents VACSGoA. Return of elevated blood pressure after withdrawal of antihypertensive drugs. *Circulation* 1975; **51:** 1107-13.

49 Wahi S, Sagar S, Sharma BK. Is a hypertensive always a hypertensive? *J Assoc Physicians India* 1993; **41:** 198-9.

Supplementary digital content 3: Changes potentially leading to adverse events

Angiotensin-II-receptor blockers

In patients with diabetes and microalbuminuria, the urinary albumin excretion rate had significantly increased by 68% at one month following the withdrawal of a low dose of Irbesartan (150mg), but the withdrawal of a high dose of Irbesartan (300mg) only led to a non-significant increase of 13% (1).

Two large cohort studies and one large cross-sectional study indicated that there is no evidence of rebound hypertension within 2 weeks of withdrawing angiotensin II receptor blockers (2-4).

Renin inhibitors

Following withdrawal of aliskiren, plasma renin activity remained reduced for up to one month (5, 6)

Angiotensin converting enzyme inhibitors

Improvements in forearm haemodynamics due to treatment with ACE inhibitors (significnat increase in brachial artery diameter and compliance, as well as a decrease in vascular resistance and pulse wave velocity) persisted at four weeks after withdrawal (values similar to those during treatment; (7)). No studies investigated pulse or heart rate following withdrawal of ACE inhibitors.

The positive effects of ACE inhibitors on renal function might reverse soon after drug cessation. Following withdrawal of ACE inhibitors, all changes due to AHT were reversed at one month including microalbuminuria and plasma N-terminal pro B-type natriuretic peptide levels (8). Anderson et al., (5) reported similar results with plasma renin activity and plasma renin concentration returning to baseline two weeks after ACE inhibitor withdrawal. People with moderate renal impairment and chronic renal failure (CRF) had no significant changes in creatinine clearance after four weeks of withdrawal of ACE inhibitors (9). However, withdrawal led to an increase of mean urinary protein, which was significant in people with CRF. In the CRF group, mean blood urea nitrogen significantly decreased following withdrawal in people who received placebos but did not decrease in people who had been treated with ACE inhibitors. For people with mild renal impairment, there was no significant difference.

Two cohort studies investigated left ventricular changes due to withdrawal of ACE inhibitors. Both studies showed that left ventricular mass did not change significantly one month after withdrawal of ACE inhibitors (8, 10). Power spectral measures of heart period variability showed that in people with LVM normalisation, the increase in day and night time high frequency powers due to treatment remained detectable at one month after withdrawal (10). However, night time total and very low frequency powers were higher after ACE inhibitor treatment compared to baseline but not at one month after withdrawal. In people without LVM normalisation due to AHT treatment, power spectral measures were lower at one month after withdrawal of ACE inhibitors compared to those with LVM normalisation.

Centrally acting drugs

Following withdrawal of alpha antagonists, small cohort studies indicated that there was no significant change in growth hormones, prolactin, insulin or glucagon after 1 week (11) and no evidence of excess catecholamine activity after 36 hours (12). A small cross-sectional study stopping beta blockers indicated a significant decrease in low density lipoproteins and triglycerides, and a significant increase in high density lipoproteins after 4 weeks of withdrawal (13).

Following withdrawal of metoprolol, no significant changes occurred in forearm haemodynamics (brachial artery circulation, vascular resistance, pulse wave velocity) at four weeks after withdrawal (7). Following the withdrawal of clonidine, pulse rate increased significantly to pre-treatment levels within two days and occasionally significantly above that during the following four days (14). Pulse rate did not raise significantly after 36 h following the withdrawal of tiamenidine (12). Two studies showed a significant increase of heart rate after the withdrawal of centrally acting agents (13, 15) and a significantly higher heart rate after withdrawal in those who had been treated with beta blockers compared to those who had been treated with calcium channel blockers (16). In addition, withdrawal from clonidine and postganglionic neurone-blocking drugs led to intense increased ectopic activity (15).

While a cross-over study reported that plasma renin activity levels overshot baseline levels following withdrawal of either Clonidine or Thiamenidine at three days after withdrawal (17), a withdrawal cohort study following the treatment arm of a RCT showed that urinary catecholamines and urinary cyclic nucleotide levels increased in the days following Clonidine withdrawal but not significantly (14). Following withdrawal of Carvedilol, changes in microalbuminuria and plasma N-terminal pro B-type natriuretic peptide levels were reversed within a month (8). Again, this suggests the positive effects of antihypertensive medications on renal function are dependent on regular medication use.

LVM did not change significantly at one month after withdrawal of beta blockers (8).

The evidence regarding rebound hypertension for centrally acting medication is mixed and based on small case, cohort and cross-over studies. Two studies showed evidence of rebound hypertension at follow-up assessments between 12 hours and two weeks after abrupt withdrawal (18, 19) and one study found systolic but no diastolic rebound hypertension at 36 hours after abrupt withdrawal of tiamenidine (12). However, Plänitz et al. (20) found no evidence for rebound hypertension after three days following abrupt withdrawal of moxonidine or clonidine.

Diuretics

Two small cohort studies and one cross sectional study indicated that stopping diuretics did not lead to a significant change in levels of growth hormones, prolactin, insulin, high density lipoproteins, triglycerides or glycohemoglobin when measured between one and 21 weeks after withdrawal (11, 13, 21). A large cohort study with a follow-up assessment at 30 months after withdrawal of HCTZ also demonstrated that there is no significant change in tryglycerides or glucose levels but the study did demonstrate significant increases in serum potassium and alkaline phosphatase as well as a significant decrease in serum uric acid (22). Furthermore, significant decreases following diuretics withdrawal were found in glucagon, total cholesterol, low density lipoproteins and the ratio of total cholesterol to HDL cholesterol when measured between one and 21 weeks after withdrawal (11, 13, 21).

The effects of thiazide diuretic withdrawal were investigated in a cohort study with a four week follow up assessment, which showed no significant change in heart rate following the withdrawal (13). No studies evaluated pulse rate or haemodynamics.

After withdrawal of hydrochlorothiazide, serum potassium levels had significantly increased and serum uric acid had significantly decreased when measured up to 30 months later (22, 23). No change in serum creatinine was found 72 weeks after withdrawal (23). The findings of a very small study (n=11; (24)) have shown that plasma renin activity rose following withdrawal of diuretics (significance level not reported) but remained constant thereafter.

Calcium antagonists

At 24 hours after withdrawal, a very small cohort study demonstrated that heart rate significantly decreased to below pre-treatment levels (25). No studies evaluated pulse rate or haemodynamics following withdrawal of calcium antagonists.

A cohort study with a very short follow-up at 24 h after Felodipine withdrawal did not show any change in plasma renin activity (25).

Vasodilators

Only one small cohort study with a follow-up assessment at 24 hours investigated rebound hypertension after abrupt withdrawal of pinacidil but the results showed no evidence of rebound hypertension (26).

Rilmenidine

After one month of withdrawal of Rilmenidine, there was no significant change in LV mass index, interventricular septum and posterior wall thickness compared to the values measured at the end of the treatment period (27). These parameters remained significantly lower after withdrawal compared to the baseline assessment before Rilmenidine treatment. LV ejection fraction did not significantly change throughout the study. LV diastolic function, which had significantly improved during treatment, returned to baseline values following withdrawal of AHT (27).

Hydrochloride

The results of a follow-up assessment two days after abrupt withdrawal of N-amidino-2- acetamide hydrochloride indicated no development of rebound hypertension (19).

Mixed or undefined AHT

Following withdrawal of AHT combination therapy, a small cohort study showed a significant decrease in total cholesterol, triglycerides and glycohemoglobin but no significant change in the ratio of total cholesterol to high density lipoprotein cholesterol when measured between 4 to 21 weeks after stopping AHT (21). In addition, a Dutch cohort study demonstrated that high cholesterol was a reason for the patient to re-start AHT even when the cardiovascular mortality risk was not considered high enough to advise AHT (28).

A risk reduction in orthostatic hypotension was demonstrated in a small cohort study including people 65 years or older (29). The percentage of people diagnosed with orthostatic hypotension significantly decreased from 23% prior to withdrawal to 11% at one year follow-up after AHT cessation.

Pulse rate was investigated in a cohort study with a 24 hour follow-up assessment (30) and heart rate was examined in a cohort study with a follow-up assessment at 2 weeks (31). Both study indicated no significant changes after withdrawal of AHT. Blood flow in response to acetylcholine has been demonstrated to be significantly higher at two weeks after AHT withdrawal than in normotensive people, but there was no significant difference in blood flow in response to nitroprusside between normotensive people and those withdrawn from AHT at two weeks (31). At 20 weeks after withdrawal, the total peripheral resistance index had raised significantly to values found in normal individuals (24). One month after withdrawal, no significant change in carotid intimal media thickness was observed (8). A randomised controlled withdrawal trial showed that post-ischaemic skin blood flow responses were significantly decreased following withdrawal in those who remained normotensive (below 140/90 mmHg) at 24 weeks after withdrawal compared to those who had continued or restarted AHT (those showed no significant change in blood flow responses;(32)).

Four cohort studies, all including a range of different AHT investigated the effects of withdrawing AHT on kidney function. A cross-sectional study investigating withdrawal of beta blockers, thiazide diuretics and hydralazine, found no significant changes in renal function when hypertension had returned, except for a significant increase in renal vascular resistance (33). Wahi et al. (34) reported that no deterioration of renal function was observed atone year after withdrawal of AHT. Two studies investigated the effects of AHT withdrawal in people with diabetic nephropathy who were insulin dependent (35) or not insulin dependent (36). In those insulin dependent, stopping AHT led to a significant increase in glomerular filtration rate (GFR) and albuminuria in 75-80% of participants at one month. In those who were not insulin dependent there was no significant change in GFR and albuminuria decreased significantly.

The majority of studies reported that there was no significant change in LV mass (37-40), LV diastolic function (37, 38, 41) or LV wall thickness (38, 41) after stopping AHT between four weeks and two years. Only one study including hemodialysis patients showed that those who re-developed hypertension after stopping AHT had a significant higher LV mass index compared to those who remained normotensive after five weeks after withdrawal (42). One study (37) showed a slight but significant decrease of LV ejection phase indices at two years after withdrawal but this was deemed as minor clinical change as 13 out of 14 patients stayed within normal range. Two further studies (38, 41)did not find a significant change in LV fractional shortening. Only one study investigated LV wall stress (37)and showed a small but significant increase after one year of withdrawal but, again, the change was deemed as a minor clinical increase as the values remained within normal limits. Furthermore, in haemodialysis patients, those remaining normotensive after AHT withdrawal at 5 weeks had developed significantly less often LV hypertrophy (50%) compared to those who had re-developed hypertension (91%).

1. Andersen S, Brochner-Mortensen J, Parving HH, Irbesartan in Patients With Type D, Microalbuminuria Study G. Kidney function during and after withdrawal of long-term irbesartan treatment in patients with type 2 diabetes and microalbuminuria. Diabetes Care. 2003;26(12):3296-302.

2. Ikeda LS, Harm SC, Arcuri KE, Goldberg AI, Sweet CS. Comparative antihypertensive effects of losartan 50 mg and losartan 50 mg titrated to 100 mg in patients with essential hypertension. Blood Press. 1997;6(1):35-43.

3. Lacourciere Y, Pool JL, Svetkey L, Gradman AH, Larochelle P, de Champlain J, et al. A randomized, double-blind, placebo-controlled, parallel-group, multicenter trial of four doses of tasosartan in patients with essential hypertension. Tasosartan Investigator's Group. Am J Hypertens. 1998;11(4 Pt 1):454-61.

4. Pool JL, Guthrie RM, Littlejohn TW, 3rd, Raskin P, Shephard AM, Weber MA, et al. Dose-related antihypertensive effects of irbesartan in patients with mild-to-moderate hypertension. Am J Hypertens. 1998;11(4 Pt 1):462-70.

5. Andersen K, Weinberger MH, Constance CM, Ali MA, Jin J, Prescott MF, et al. Comparative effects of aliskiren-based and ramipril-based therapy on the renin system during long-term (6 months) treatment and withdrawal in patients with hypertension. J Renin Angiotensin Aldosterone Syst. 2009;10(3):157-67.

6. Schneider MP, Janka R, Ziegler T, Raff U, Ritt M, Ott C, et al. Reversibility of the effects of aliskiren in the renal versus systemic circulation. Clin J Am Soc Nephrol. 2012;7(2):258-64.

7. De Cesaris R, Ranieri G, Filitti V, Andriani A, Bonfantino MV. Forearm arterial distensibility in patients with hypertension: comparative effects of long-term ACE inhibition and beta-blocking. Clin Pharmacol Ther. 1993;53(3):360-7.

8. Saul SM, Duprez DA, Zhong W, Grandits GA, Cohn JN. Effect of carvedilol, lisinopril and their combination on vascular and cardiac health in patients with borderline blood pressure: the DETECT Study. J Hum Hypertens. 2013;27(6):362-7.

9. Miller MA, Texter M, Gmerek A, Robbins J, Shurzinske L, Canter D. Quinapril hydrochloride effects on renal function in patients with renal dysfunction and hypertension: a drug-withdrawal study. Cardiovasc Drugs Ther. 1994;8(2):271-5.

10. Petretta M, Bonaduce D, Marciano F, Bianchi V, Valva G, Apicella C, et al. Effect of 1 year of lisinopril treatment on cardiac autonomic control in hypertensive patients with left ventricular hypertrophy. Hypertension. 1996;27(3 Pt 1):330-8.

11. Eldridge JC, Strandhoy J, Buckalew VM, Jr. Endocrinologic effects of antihypertensive therapy with guanabenz or hydrochlorothiazide. J Cardiovasc Pharmacol. 1984;6 Suppl 5:S776-80.

12. Gerlis LS, Wright N. Abrupt withdrawal of the antihypertensive agent tiamenidine after 12-months' treatment. Pharmatherapeutica. 1980;2(6):408-11.

13. Middeke M, Richter WO, Schwandt P, Beck B, Holzgreve H. Normalization of lipid metabolism after withdrawal from antihypertensive long-term therapy with beta blockers and diuretics. Arteriosclerosis. 1990;10(1):145-7.

14. Nami R, Bianchini C, Fiorella G, Chierichetti SM, Gennari C. Comparison of effects of guanfacine and clonidine on blood pressure, heart rate, urinary catecholamines, and cyclic nucleotides during and after administration to patients with mild to moderate hypertension. J Cardiovasc Pharmacol. 1983;5(4):546-51.

15. Goldberg AD, Raftery EB, Wilkinson P. Blood pressure and heart rate and withdrawal of antihypertensive drugs. British Medical Journal. 1977;1(6071):1243-6.

16. Schmieder RE, Ruddel H, Neus H, Neus J, von Eiff AW. Predictors of blood pressure increases after withdrawal of antihypertensive therapy. J Hypertens Suppl. 1985;3(3):S457-9.

17. Hansson BG, Hokfelt B. Changes in blood pressure, plasma catecholamines and plasma renin activity during and after treatment with tiamenidine and clonidine. Br J Clin Pharmacol. 1981;11(1):73-7.

18. Bruce DL, Croley TF, Lee JS. Preoperative clonidine withdrawal syndrome. Anesthesiology. 1979;51(1):90-2.

19. Kirch W, Distler A. Antihypertensive effect of N-amidino-2-(2,6-dichlorophenyl) acetamide hydrochloride. A double-blind cross-over trial versus clonidine. Int J Clin Pharmacol Biopharm. 1978;16(3):132-5.

20. Planitz V. Crossover comparison of moxonidine and clonidine in mild to moderate hypertension. Eur J Clin Pharmacol. 1984;27(2):147-52.

21. Ames RP, Hill P. Improvement of glucose tolerance and lowering of glycohemoglobin and serum lipid concentrations after discontinuation of antihypertensive drug therapy. Circulation. 1982;65(5):899-904.

22. Freis ED, Thomas JR, Fisher SG, Hamburger R, Borreson RE, Mezey KC, et al. Effects of reduction in drugs or dosage after long-term control of systemic hypertension. Am J Cardiol. 1989;63(11):702-8.

23. Agents VACSGoA. Return of elevated blood pressure after withdrawal of antihypertensive drugs. Circulation. 1975;51(6):1107-13.

24. Jennings GL, Korner PI, Laufer E, Esler MD, Burton D, Bruce A. How hypertension redevelops after cessation of long-term therapy. J Hypertens Suppl. 1984;2(3):S217-9.

25. Smith SA, Mace PJ, Littler WA. Felodipine, blood pressure, and cardiovascular reflexes in hypertensive humans. Hypertension. 1986;8(12):1172-8.

26. Caruana MP, Al-Khawaja I, Royston P, Raftery EB. The effects of long-acting pinacidil on intra-arterial blood pressure. Br J Clin Pharmacol. 1985;20(2):140-3.

27. Trimarco B, Rosiello G, Sarno D, Lorino G, Rubattu S, DeLuca N, et al. Effects of one-year treatment with rilmenidine on systemic hypertension-induced left ventricular hypertrophy in hypertensive patients. Am J Cardiol. 1994;74(13):36A-42A.

28. van Duijn HJ, Belo JN, Blom JW, Velberg ID, Assendelft WJ. Revised guidelines for cardiovascular risk management - time to stop medication? A practice-based intervention study. Br J Gen Pract. 2011;61(587):e347-52.

29. Fotherby MD, Potter JF. Orthostatic hypotension and anti-hypertensive therapy in the elderly. Postgrad Med J. 1994;70(830):878-81.

30. Byyny RL, Merrill DD, Bradstreet TE, Sweet CS. An inpatient trial of the safety and efficacy of losartan compared with placebo and enalapril in patients with essential hypertension. Cardiovasc Drugs Ther. 1996;10(3):313-9.

31. Panza JA, Quyyumi AA, Callahan TS, Epstein SE. Effect of antihypertensive treatment on endothelium-dependent vascular relaxation in patients with essential hypertension. J Am Coll Cardiol. 1993;21(5):1145-51.

32. Sieg-Dobrescu D, Burnier M, Hayoz D, Brunner HR, Waeber B. The return of increased blood pressure after discontinuation of antihypertensive treatment is associated with an impaired post-ischemic skin blood flow response. J Hypertens. 2001;19(8):1387-92.

33. Ljungman S, Aurell M, Hartford M, Wikstrand J, Berglund G. Renal function before and after withdrawal of long term antihypertensive treatment in primary hypertension. Drugs. 1988;35 Suppl 5:55-8.

34. Wahi S, Sagar S, Sharma BK. Is a hypertensive always a hypertensive? J Assoc Physicians India. 1993;41(4):198-9.

35. Hansen HP, Rossing P, Tarnow L, Nielsen FS, Jensen BR, Parving HH. Increased glomerular filtration rate after withdrawal of long-term antihypertensive treatment in diabetic nephropathy. Kidney Int. 1995;47(6):1726-31.

36. Hansen HP, Nielsen FS, Rossing P, Jacobsen P, Jensen BR, Parving HH. Kidney function after withdrawal of long-term antihypertensive treatment in diabetic nephropathy. Kidney Int Suppl. 1997;63:S49-53.

37. Lernfelt B, Landahl S, Svanborg A, Wikstrand J. Overtreatment of hypertension in the elderly? J Hypertens. 1990;8(5):483-90.

38. Muiesan ML, Salvetti M, Monteduro C, Rizzoni D, Corbellini C, Castellano M, et al. Changes in midwall systolic performance and cardiac hypertrophy reduction in hypertensive patients. J Hypertens. 2000;18(11):1651-6.

39. Myers MG, Reeves RA, Oh PI, Joyner CD. Overtreatment of hypertension in the community? Am J Hypertens. 1996;9(5):419-25.

40. Prasad N, Davey PG, Watson AD, Peebles L, MacDonald TM. Safe withdrawal of monotherapy for hypertension is poorly effective and not likely to reduce health-care costs. J Hypertens. 1997;15(12 Pt 1):1519-26.

41. Perlini S, Muiesan ML, Cuspidi C, Sampieri L, Trimarco B, Aurigemma GP, et al. Midwall mechanics are improved after regression of hypertensive left ventricular hypertrophy and normalization of chamber geometry. Circulation. 2001;103(5):678-83.

42. Bishu K, Gricz KM, Chewaka S, Agarwal R. Appropriateness of antihypertensive drug therapy in hemodialysis patients. Clin J Am Soc Nephrol. 2006;1(4):820-4.
